# Assessing levels and trends of child health inequality in 88 developing countries: from 2000 to 2014

**DOI:** 10.1080/16549716.2017.1408385

**Published:** 2017-12-13

**Authors:** Zhihui Li, Mingqiang Li, S. V. Subramanian, Chunling Lu

**Affiliations:** ^a^ Department of Global Health and Population, Harvard T.H Chan School of Public Health, Boston, MA, USA; ^b^ Department of Social and Behavioral Sciences, Harvard T.H. Chan School of Public Health, Boston, MA, USA; ^c^ Department of Medicine, Brigham & Women’s Hospital/Harvard Medical School, Boston, MA, USA; ^d^ Department of Science and Technology-National Research Foundation (DST-NRF) Center of Excellence in Human Development, University of Witwatersrand, Johannesburg, South Africa

**Keywords:** Child health interventions, child mortality, inequality, child health outcomes, developing countries

## Abstract

**Background**: Reducing child mortality was one of the Millennium Development Goals. In the current Sustainable Development Goals era, achieving equity is prioritized as a major aim.

**Objective**: This study aims to provide a comprehensive and updated picture of inequalities in child health intervention coverage and child health outcomes by wealth status, as well as their trends between 2000 and 2014.

**Methods**: Using data from Demographic Health Surveys and Multiple Indicator Cluster Surveys, we adopted three measures of inequality, including one absolute inequality indicator and two relative inequality indicators, to estimate the level and trends of inequalities in three child health outcome variables and 17 intervention coverages in 88 developing countries.

**Results**: While improvements in child health outcomes and coverage of interventions have been observed between 2000 and 2014, large inequalities remain. There was a high level of variation between countries’ progress toward reducing child health inequalities, with some countries significantly improving, some deteriorating, and some remaining statistically unchanged. Among child health interventions, the least equitable one was access to improved sanitation (The absolute difference in coverages between the richest quintile and the poorest quintile reached 49.5% [42.7, 56.2]), followed by access to improved water (34.1% [29.5, 38.6]), and skilled birth attendant (SBA) (34.1% [28.8, 39.4]). The most equitable intervention coverage was insecticide-treated bed net for children (1.0% [−3.9, 5.9]), followed by oral rehydration therapy for diarrhea ((8.0% [5.2, 10.8]), and vitamin A supplement (8.4% [5.1, 11.7]). These findings were robust to various inequality measurements.

**Conclusions**: Although child health outcomes and coverage of interventions have improved largely over the study period for almost all wealth quintiles, insufficient progress was made in reducing child health inequalities between the poorest and richest wealth quintiles. Future efforts should focus on reaching the poorest children by increasing investments toward expanding the coverage of interventions in resource-limited settings.

## Background

Reducing child mortality was one of the Millennium Development Goals (MDGs). Since 1990, worldwide under-five mortality has dropped from 91 deaths per 1,000 live births in 1990 to 43 in 2015 [1]. Despite this apparent reduction in the under-five mortality rate, the national-level aggregate data often obscure the fact that the poorest global populations have not experienced nearly such improvements [2]. It is estimated that children in the poorest households were three times more likely to die before the age of five than were those in the richest quintile. This trend remained constant in some countries, but worsened in the majority of them [3,4]. The Sustainable Development Goals (SDGs) prioritize improving equity as a major goal for the period 2015–2030 and emphasize reducing child health inequalities both within and between countries [5].

A large amount of the previous literature on child health equity in developing countries has focused on coverage of interventions in the health sector of selected countries such as Brazil, Vietnam, China, Thailand, and Tanzania [6–10]. These studies provided firm evidence that socioeconomic status is an essential factor associated with child health. Studies by Barros AJ et al., Victora CG et al., and the Countdown 2008 equity analysis group have investigated the levels and trends of socioeconomic inequality in maternal and child health interventions in the Countdown countries (a maximum of 54 Countdown countries) (Countdown countries—a group of high-burden priority countries, which together represent 95% of maternal and child mortality [11]) between 1990 and 2008 [12–14]. These studies showed that, although there was noticeable improvement in child health interventions, inequalities persisted in many countries. Since 2010, the Countdown group has reported detailed equity profiles for each Countdown country [15].

Our study aims to assess the level and trends of child health inequalities by wealth status in 88 developing countries with available data from 2000 to 2014. Our study will expand upon previous analyses by widening the scope of the countries from 54 to 88 developing countries, including both Countdown and non-Countdown countries. The time frame is also extended from 2000–2014, with the most updated data. In addition, we include more indicators to measure child health inequalities than have previous studies. For example, we tracked inequalities in three child health outcome indicators (infant mortality, under-five mortality, and stunting). Previous studies analyzed inequality in coverage for 12 child health interventions [12,16–18]; we added an additional five interventions in our study, including access to improved water, access to improved sanitation, Bacillus Calmette–Guérin (BCG) immunization, polio immunization, and care seeking for diarrhea. To our knowledge, this is the first study that provides a comprehensive picture of the progress made in reducing inequalities in child health outcomes and across 17 child health interventions for 88 countries between 2000 and 2014.

## Methods

### Data sources and procedures

Our primary data sources are the Demographic Health Surveys (DHS) and Multiple Indicator Cluster Surveys (MICS) conducted between 2000 and 2014. The DHS and MICS data are highly comparable, as their technical teams have collaborated closely and work together through interagency processes to ensure that survey tools are harmonized [19]. We excluded interim DHS surveys due to their relatively small sample size and limited available indicators [20]. We identified 88 developing countries with available DHS or MICS data subsequent to 2000. Following previous studies [13,14], we divided the surveys into three rounds according to the date of their implementation: round 1 for 2000–2004, round 2 for 2005–2009, and round 3 for 2010–2014. Of the 88 countries, three conducted their latest surveys in survey round 1, 20 in survey round 2, and 65 in survey round 3. (Table A1) The median of the latest survey year is 2011, with an interquartile range between 2009 and 2013.

**Table 1.** Mean values of absolute inequalities, with 95% confidence interval, of three child health outcomes and 10 child health interventions, most recent survey data for 88 countries.

					Difference in Q5 – Q1			
	No. of countries	Overall value	Q1	Q5	Mean	IQR	Bottom decile (10%)	Top decile (10%)
**Child health outcomes**								
Infant mortality per 1,000 live births	52	50.4 [44.8, 56.0]	58.9 [52.6, 65.3]	36.3 [31.3, 41.4]	−22.6 [−26.8, −18.3]	19.2	−45.2	−2.0
Under-five mortality per 1,000 live births	52	76.0 [65.6, 86.4]	91.8 [79.8, 103.8]	49.5 [41.5, 57.5]	−42.3 [−49.5, −35.1]	31.0	−77.3	−16.4
Stunting prevalence (%)	80	28.0% [25.1, 30.8]	35.4% [32.1, 38.7]	17.2% [14.8, 19.5]	−18.2% [−20.5, −15.9]	13.8%	−34.5%	−4.8%
**Child health interventions**								
Access to improved sanitation (%)	83	59.5% [53.0, 65.9]	38.2% [30.3, 46.2]	87.8% [83.6, 91.9]	49.5% [42.7, 56.2]	62.6%	5.3%	89.1%
Access to improved water (%)	83	75.5% [71.9, 79.1]	58.9% [53.5, 64.3]	93.0% [91.3, 94.7]	34.1% [29.5, 38.6]	35.6%	5.6%	62.5%
Skilled birth attendant (%)	88	74.0% [69.2, 78.8]	58.8% [52.3, 65.4]	92.9% [91.0, 94.9]	34.1% [28.8, 39.4]	44.7%	0.5%	68.6%
Four or more antenatal care visits (%)	64	61.1% [55.7, 66.5]	49.7% [43.2, 56.1]	77.7% [73.5, 81.8]	27.9% [23.3, 32.6]	28.1%	3.2%	53.6%
Full immunization (%)	84	62.4% [57.8, 67.0]	55.5% [50.0, 60.9]	68.5% [64.2, 72.8]	13.0% [9.3, 16.7]	25.0%	−8.0%	33.8%
ORT for diarrhea (%)	85	40.4% [36.5, 44.4]	37.0% [32.9, 41.1]	44.6% [40.8, 48.3]	8.0% [5.2, 10.8]	16.7%	−5.5%	24.6%
Care seeking for suspected pneumonia (%)	87	59.5% [54.6, 64.3]	52.4% [47.2, 57.6]	70.3% [66.1, 74.6]	18.8% [15.4, 22.2]	22.4%	3.0%	36.5%
Vitamin A supplement (%)	64	53.8% [48.5, 59.1]	49.6% [44.2, 55.0]	58.0% [52.6, 63.5]	8.4% [5.1, 11.7]	17.7%	−8.5%	23.5%
ITN for children (%)	38	38.4% [32.3, 44.6]	36.1% [29.9, 42.2]	37.0% [30.2, 43.9]	1.0% [−3.9, 5.9]	17.1%	−16.9%	22.1%

The method used for the calculations in this table is presented in the ‘Appendix Method’ section.

Among the 88 countries, 41 had undertaken all three rounds of surveys, enabling us to analyze the trends of inequality in child health outcomes and interventions with available indicators. For countries with multiple years of data available within one survey round, we used data from the most recent year available, so as to avoid overweighting such countries in our analysis.

To avoid repeating previous work on measuring indicators by wealth quintiles, we utilized the WHO Global Health Observatory (GHO) database [21]. Using the DHS and MICS surveys, the GHO provides calculated data by wealth quintile for a group of indicators of child health outcomes and child health interventions, such as infant mortality, under-five mortality, and SBA. One issue in using the GHO database is that estimates are only available until 2012, and data from earlier years (2000–2004) or after 2012 in some countries are unrecorded. Therefore, we manually estimated values of health indicators by wealth quintiles for these years following the method adopted by the WHO GHO [22]. Table A2 lists the data from GHO and estimates from our own calculations.

### Selection of child health indicators

#### Indicators on child health outcomes

We selected three child health outcome indicators, infant mortality, under-five mortality, and stunting prevalence. We chose these indicators for the following reasons: reducing under-five mortality (including infant mortality) is one of the health-related MDGs that have been the focus of global health agendas [23]. Stunting is a cause of public concern in many developing countries, and reducing stunting prevalence is one of the key components of improving child health. These three indicators have been commonly used in previous literatures for assessing the health status of children (Table A3) [7,9,24,25].

#### Indicators on child health interventions

We included 17 interventions that collectively account for all stages of the continuum of care for child health. Twelve of these were included in previous studies (Table A3) for their well-documented effects on child mortality and health [12,16–18]. We added five additional interventions that have demonstrated a significant impact on child health and have available data in DHS and MICS: access to improved water, access to improved sanitation, BCG immunization, polio immunization, and care seeking for diarrhea [9,26–31].

Notably, the wealth index variable in DHS and MICS, which categorizes households into five wealth quintiles, is calculated using ownership of selected assets, such as televisions and bicycles; materials used for housing construction, and types of water access and sanitation facilities. When analyzing the inequality of access to improved water or sanitation, we followed the DHS guideline on constructing wealth indexes and produced a new set of wealth indexes that do not include water sources and sanitation facilities [32,33].

### Measurements of inequality

Following previous studies [12,13], we adopted the three most commonly used inequality measurements, including one indicator for absolute inequality (the difference of the indicators between the richest wealth quintile, ‘Q5’, and poorest wealth quintile, ‘Q1’) and two indicators for relative inequality (the ratio of Q5 to Q1 and concentration index).

For each indicator at the country level, we generated values for each wealth quintile (Q1 to Q5). We calculated the absolute differences between Q1 and Q5 to assess the indicator’s absolute inequality status, and the ratio of Q5 to Q1 to assess the indicator’s relative inequality status.

We generated concentration indexes from the concentration curves that plot the cumulative proportion of one variable against the cumulative proportion of the population ranked by wealth. Concentration indexes capture the extent to which health outcomes/health interventions differ across individuals’ ranks by wealth. A detailed calculation method for concentration indexes is referred to in the World Bank’s instructions [34]. The concentration index is expressed on a scale ranging from −100 to 100, with zero representing perfect equality. If the health indicator is undesirable, such as infant mortality, a negative value of the concentration index means that ill health is more prevalent among the poor. The more negative a concentration index, the more inequality it suggests. If the health outcome is desirable, a positive value of concentration index indicates the health of the rich is better than the health of the poor. The more positive a concentration index, the more inequality it suggests. Calculations, features, and limitations of the three measurements are presented in Table A4.

To provide a more comprehensive picture of inequality, we also included the interquartile range (IQR), bottom decile, and top decile for the three inequality measures. We calculated the 95% confidence intervals (CIs) for Q1, Q5, and three inequality measures. The calculation methods of the 95% CIs are presented in the Appendix Method section.

This study focused on the absolute inequality between Q1 and Q5, as it represents the most intuitive among all inequality measurements. By showing the scope of the gaps, policymakers and the general public can easily understand by how much the poorest population lags behind the richest [35].

### Inequality analysis

When conducting trend analysis of an indicator, we consider it essential to compare the same group of countries over time. In order to achieve it, we only included the countries with valid data on an indicator in all three survey rounds. For example, 34 countries had a variable indicating child stunting prevalence in all three rounds. When analyzing the inequality of child stunting prevalence over time, we only included these 34 countries in the analysis. Table A5 contains data availability for each indicator.

To determine whether the inequality status of an indicator at either aggregate or country level changed significantly over time, we compared the 95% CI of the indicator in each available round. If the 95% CIs of the indicator in the two rounds did not overlap, we concluded that there was a significant change over time, or vice versa. We are aware that there are cases when the 95% CIs do not overlap, yet the differences are still significant, so we also conducted a *t*-test for the nonoverlapping values. STATA 14 was used in analysis.

## Results

*Mean* *level of inequality status in child health outcomes and interventions for 88 countries, most recent years*

Table 1 presents the mean level of absolute inequality in three child health outcomes and nine selected child health interventions, using data from the most recent survey of each country. Full tables with 17 interventions are presented in Tables A6 **and A7**. Except for the insecticide-treated bed net (ITN) for children, the difference between Q5 and Q1 for all indicators differed significantly from zero: a result smaller than zero for outcome indicators and a result larger than zero for intervention indicators. This suggests that children in the richest quintile had better health outcomes and higher coverage rates of interventions than did children in the poorest quintile across countries.

The mean differences between Q5 and Q1 for the 52 countries in infant and under-five mortality were −22.6 [−26.8, −18.3] and −42.3 [−49.5, −35.1] respectively. The mean difference between Q5 and Q1 in stunting prevalence for 80 countries was −18.2% [−20.5, −15.9]. Among the 80 countries, 34 countries had a high stunting prevalence (≥30%), and the mean difference between Q5 and Q1 in these countries was −22.2% [−25.3, −19.1].

The intervention with the largest inequality was access to improved sanitation (49.5% [42.7, 56.2]), followed by access to improved water (34.1% [29.5, 38.6]), SBA (34.1% [28.8, 39.4]), and four or more antenatal care visits (27.9% [23.3, 32.6]). The most equitable coverage of intervention was ITN for children (1.0% [−3.9, 5.9]), followed by oral rehydration therapy (ORT) for diarrhea (8.0% [5.2, 10.8]) and vitamin A supplementation (8.4% [5.1, 11.7]). These results remain consistent over all three inequality measurements. (Table A6)

*Trends of mean inequality status in child health outcomes and child health interventions: round 1 vs. round 3*

**Figure 1.**
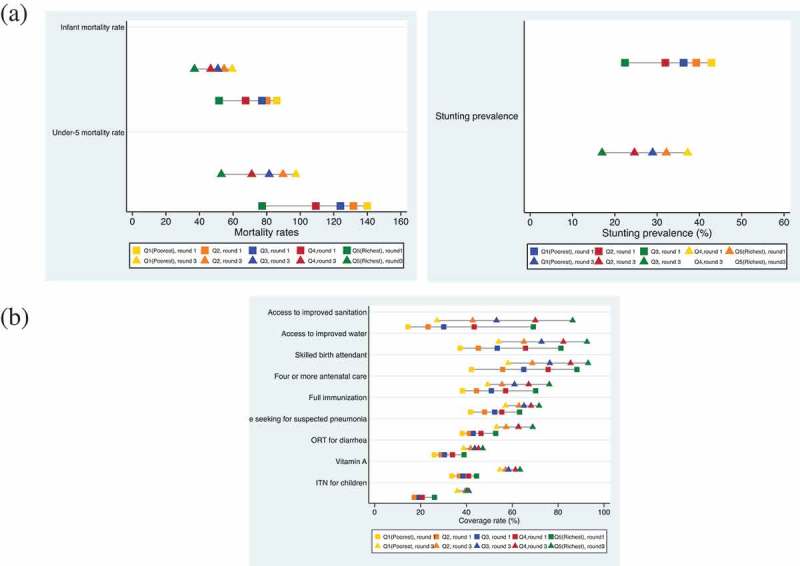
(a) Prevalence of child health outcomes in each wealth quintile, round 1 vs. round 3; various numbers of countries were involved in analyzing different indicators. (b) Coverage rates of nine selected child health interventions in each wealth quintile, round 1 vs. round 3; various numbers of countries were involved in analyzing different indicators. The interventions are listed according to the sum of the absolute differences between Q5 and Q1 in round 1 and round 3: ‘Access to improved sanitation’ is with the largest summed difference and ‘ITN for children’ is with the smallest summed difference.

Figure 1(a and b) shows the mean prevalence of the three child health outcomes and the coverage rates of nine selected child health interventions by wealth quintile in round 1 and round 3. A complete figure, with eight additional interventions, is presented in Appendix Figure 1.

Figure 1(a) shows in each wealth quintile that the mean infant mortality rate and under-five mortality rate reduced substantially over time. For example, the under-five mortality of the poorest quintile decreased from 139.9 per 1,000 live births in round 1 to 97.4 per 1,000 live births in round 3. The mean difference between Q5 and Q1 also largely shrank; the difference in infant mortality decreased from 34.4 per 1,000 live births in round 1 to 22.4 in round 3; the difference in under-five morality decreased from 62.6 per 1,000 live births in round 1 to 44.4 in round 3. The mean stunting prevalence, however, experienced a much smaller reduction between round 1 and round 3. For example, the stunting prevalence for the poorest quintile was 42.8% in round 1 and was only slightly reduced to 41.4% in round 3. The mean difference between Q5 and Q1 also remained large and did not improve over time, moving from 20.4% in round 1 to 20.2% in round 3.

We observed that the coverage rates of all nine interventions increased between round 1 and round 3 in each wealth quintile for countries with available data in round 1 and round 3 (Figure 1(b)). Consistent with results presented in Table 1, we found that access to improved sanitation had the largest difference between Q5 and Q1, 54.7% in survey round 1 and 59.1% in survey round 3. Other indicators that rank highly for mean inequality include supply of improved water, SBA, and four or more antenatal care visits: the differences between Q5 and Q1 for these three indicators were 46.0%, 44.0%, and 31.9% respectively in round 1, and decreased to 34.9%, 38.1%, and 26.5% in round 3. Except for access to improved sanitation and care seeking for suspected pneumonia, seven out of nine selected indicators showed reductions in inequalities between round 1 and round 3.

*Country-level change in inequality status in child health outcomes and child health interventions: round 1 vs. round 3*

**Figure 2.**
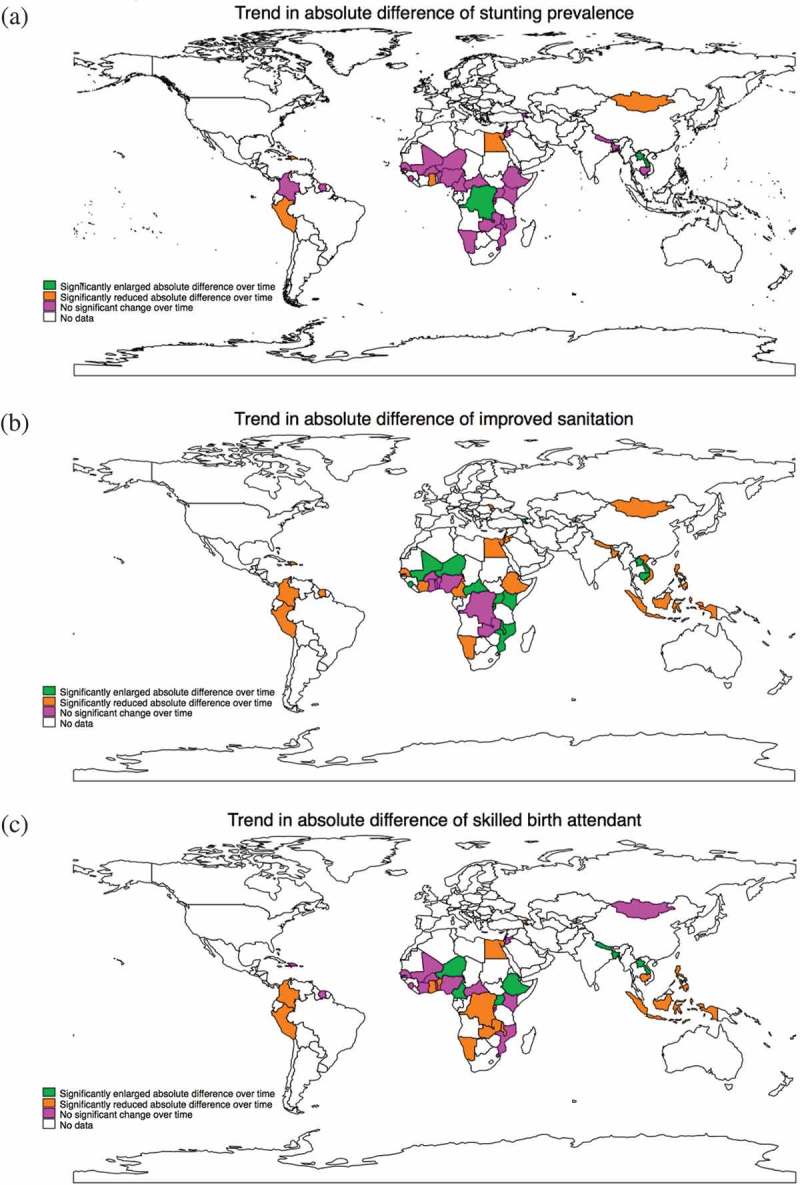
(a) World map on the change of absolute difference between Q5 and Q1 in stunting prevalence, round 1 vs. round 3. (b) World map on the change of absolute difference between Q5 and Q1 in access to improved sanitation, round 1 vs. round 3. (c) World map on the change of absolute difference between Q5 and Q1 in skilled birth attendant, round 1 vs. round 3.

**Figure A2.**
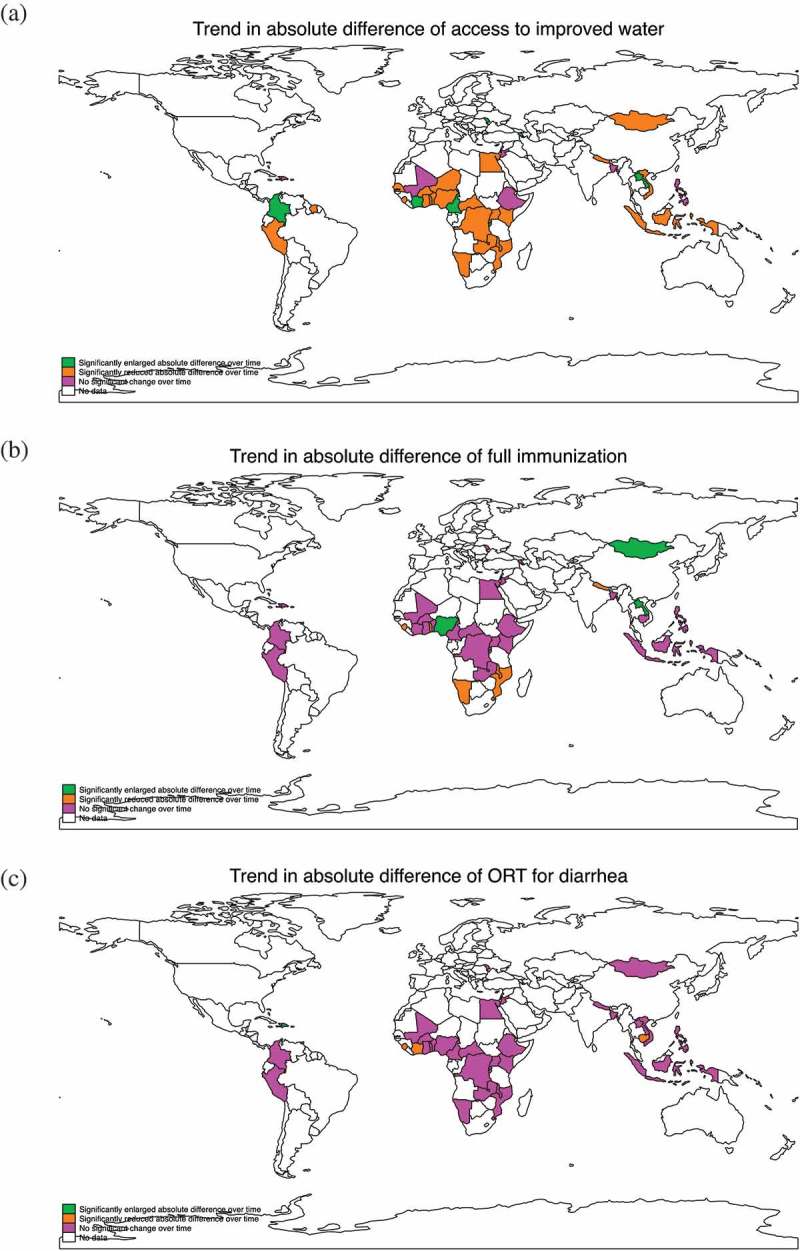
**(a)** World map on the change of absolute difference between Q5 and Q1 in access to improved water, round 1 vs. round 3; **(b)** World map on the change of absolute difference between Q5 and Q1 in coverage of full immunization, round 1 vs. round 3; **(c)** World map on the change of absolute difference between Q5 and Q1 in ORT for diarrhea, round 1 vs. round 3.

Figure 2 shows the changes in absolute inequalities in three indicators that have data available in all three rounds in more than 30 countries: stunting prevalence, access to improved sanitation, and SBA. Figure A2 further shows the change in three more indicators with data available in more than 30 countries, including access to improved water, coverage of full immunization, and ORT for diarrhea. Tables A8 **and A9** present the inequality status of the indicators in each round for each country.

In Figure 2(a), we observed that among the 34 countries with valid data on child stunting status in all three rounds, five of them experienced a significant reduction in the difference between Q5 and Q1 during survey rounds 1 and 3, including Dominican Republic, Egypt, Ghana, Mongolia, and Peru (marked in orange in Figure 2(a)); three of them were Countdown countries. Two countries (Congo, Dem. Rep., and Lao PDR, marked in green in Figure 2(a)) experienced significantly enlarged inequality in stunting prevalence between rounds 1 and 3. Strikingly, the inequality in stunting prevalence between Q5 and Q1 in Laos increased from −12.7% [−19.1, −6.4] in round 1 to −40.9% [−44.3, −37.4] in round 3, indicating a severe deterioration in the equitability of child stunting. In round 1, Peru possessed the largest absolute difference between Q5 and Q1 (−49.5% [−54.9, −44.0]), followed by Nigeria −33.0% [−39.2, −26.7]) and Bangladesh (−31.6% [−36.3, −26.8]). In round 3, stunting prevalence was significantly reduced to −35.0% [−38.1, −31.8] in Peru and remained statistically unchanged in Nigeria and Bangladesh.

As shown in Figure 2(b), of the 40 countries with available data on access to improved sanitation, 18 significantly reduced the difference between Q5 and Q1; five of them were located in Sub-Saharan Africa (Cameroon, Cote d’Ivoire, Ethiopia, Namibia, and Senegal); four in East Asia & Pacific (Mongolia, Philippines, Vietnam, and Indonesia); and four in Latin America & Caribbean (Colombia, Dominican Republic, Peru, and Suriname). The remaining five were scattered across South Asia (Bangladesh and Nepal), Middle East & North Africa (Egypt and Jordan), and Europe & Central Asia (Moldova). Sixteen of the 18 countries were Countdown countries. Nine of them reduced the difference between Q5 and Q1 by more than 20 percentage points, including Dominican Republic, Ethiopia, Jordan, Moldova, Mongolia, Nepal, Peru, Suriname, and Vietnam. However, 14 countries experienced significant increases in the absolute difference, (marked in green in Figure 2(b)) with 10 of them representing Sub-Saharan African countries. Thirteen of them were Countdown countries.

Figure 2(c) shows that, among the 39 countries with available data on delivery with SBA in all three rounds, 14 experienced a significant reduction in the difference between Q5 and Q1 between round 1 and round 3, including seven countries in Sub-Saharan African (Benin, Congo, Dem. Rep., Ghana, Malawi, Namibia, Rwanda, and Zambia), three countries in East Asia & Pacific (Cambodia, Indonesia, and Philippine), and two countries in Latin America & Caribbean (Colombia and Peru). Eleven of the 14 are Countdown countries. Seven of them reduced the difference between Q5 and Q1 by more than 20 percentage points, including Cambodia, Egypt, Ghana, Malawi, Peru, Rwanda, and Zambia. On the other hand, seven countries showed a significantly enlarged difference between Q5 and Q1 in SBA (marked in green in Figure 2(c)): four countries located in Sub-Saharan Africa (Cameroon, Ethiopia, Niger, and Uganda); two located in South Asia (Bangladesh and Nepal); and one in East Asia & Pacific (Lao PDR). All of them were Countdown countries. The estimates of relative inequality (Tables A10–A13) were basically aligned with the estimates of absolute inequality.

## Discussion and conclusion

This study has two salient findings. Firstly, remarkable improvements in child health and coverage of interventions have been observed between 2000 and 2014 in both Q1 and Q5, yet large inequalities remain in these indicators. Except for ITN, the mean differences between Q5 and Q1 for all other indicators were significantly different from zero in the most recent survey round.

Though analyzing determinants of inequality is beyond the scope of this study, we speculate that measuring the progress of reaching MDGs by focusing on national means could lead to less policy attention paid to inequalities [36]. A 2010 UNICEF report found that progress measured by national aggregates often conceals large and widening disparities in child health—despite apparent statistical success in reducing under-five mortality, inequalities between the poorest and the richest households grew by more than 10% [37]. As the Countdown equity analysis indicates, child health interventions tend to reach the wealthiest children first in the absence of policy instruments for addressing inequality [14]. The second salient finding is that the progress made in reducing child health inequalities differs greatly by country, with some countries making significant improvements between 2000 and 2014, some significantly deteriorating, and others remaining statistically unchanged. For example, we found that 18 countries significantly reduced the difference between Q5 and Q1 in access to improved sanitation over time, with nine of them reducing the difference by more than 20 percentage points, indicating a remarkable improvement in equality of access to improved sanitation. Meanwhile, we observed a significant growth in absolute difference in 14 countries, implying a deteriorating level of equality in access to improved sanitation.

Although significant reductions or increases in child health inequalities have occurred in both Countdown and non-Countdown countries, we found that the deterioration in inequality was heavily concentrated in Countdown countries. All countries experiencing deteriorating inequality in child health outcomes were Countdown countries. Among those countries with deteriorating inequality in the coverage of child health interventions, more than 90% were Countdown countries. The following may be plausible explanations for this situation: firstly, out-of-pocket medical payments for receiving care could be too high for the poorest households in Countdown countries, thus deterring their access to the related care. For example, in Ethiopia, delivery at health institutions costs $17–22, while home delivery with traditional birth attendants costs only $0.8. Consequently, poor families may choose not to use SBA during deliveries [38]. Secondly, travel costs and foregone earnings are important opportunity costs in consuming health services for the poorest households, especially those living in remote rural areas. In Uganda, for example, 55.4% of the poorest population (the majority of which reside in rural areas) considered the long distance to a health facility a barrier to accessing health services, compared with 17.2% in the richest population [39]. Thirdly, poor quality of care could prevent poor rural patients in countries such as Rwanda from using the services [40]. It is estimated that, in rural Bangladesh, the absentee rate for physicians is 40% at larger clinics and 74% at smaller subcenters with a single physician. The number of patients was found to be negatively correlated with absentee rate [41]. Other factors, such as lack of proper knowledge/education on illness, lack of awareness of exemption of or subsidy to medical care spending for the poor, and cultural and social norms could also contribute to these inequalities [42–44].

This paper provides two distinct contributions to the study of child health inequalities. Firstly, taking advantage of available data, we extended the previous analyses by expanding the scope of considered countries (88 countries with 57 Countdown and 31 non-Countdown countries) and time (between 2000 and 2014). Secondly, we expanded upon the inequalities studied by including three child health outcomes and five interventions, such as access to improved water and sanitation.

There are several limitations to this study. Firstly, the classification of wealth quintiles is country-specific and time-sensitive. The poorest quintile in an upper-middle-income country could be better off than the richer quintiles in a less developed country. As a country’s economy grows, the definition of the poorest quintile may also change [12,32]. Secondly, due to the varying availability of variables in the DHS and MICS by country and year, we were only able to conduct a trend analysis for a subset of countries, which limited our knowledge on the progress of reducing inequality in all 88 countries.

Moving forward, updating assessments on child health inequality, both within and across countries, is essential as more data become available. More research needs to be conducted to identify the facilitators or barriers to reducing inequality in child health. For example, similar to Barros et al.’s findings [12], our study shows that interventions that had to be delivered at health institutions, particularly those that need access to secondary-level or tertiary-level care, such as SBA, were among the least equitable. Interventions that could be delivered at the community level, such as vitamin A supplements, tend toward greater equitability [45]. This may suggest that expanding community health programs could be positively linked to the reduction of inequality in intervention coverage. In addition, improving equality should be prioritized at the national and global health agendas. In the SDG era, it is critical to monitor progress in child health, to focus not only on population means but also on inequalities.

## Supplementary Material

Supplementary material

## Figures and Tables

**Figure A1.**
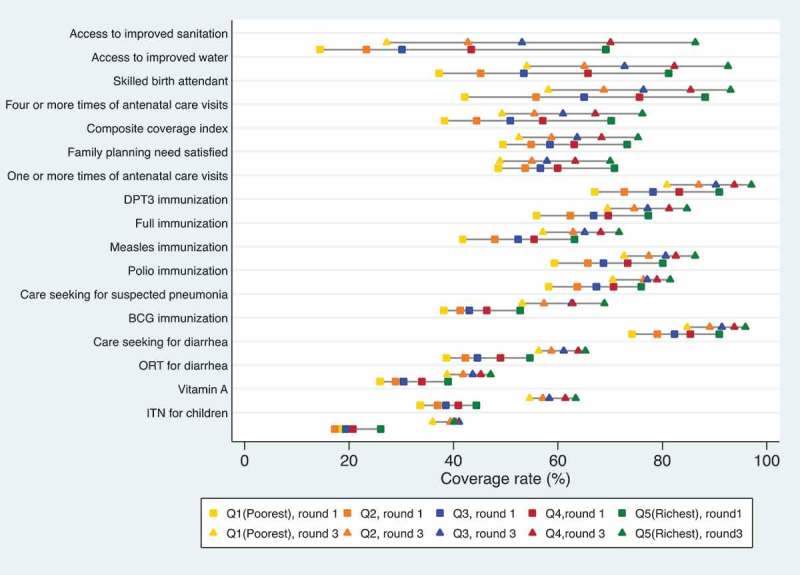
Coverage of 17 child health interventions in each wealth quintile, round 1 vs. round 3. Various numbers of countries were involved in analyzing different indicators.

## Paper context

While previous studies on child health inequalities exclusively focused on interventions in the health sector, we yielded a comprehensive evaluation of child health, tracking inequalities in three outcome indicators (mortality and stunting) and 17 interventions including access to improved water and sanitation. We also extended the previous analyses by expanding the scope of the countries (88 countries), and time length (2000–2014). We call for greater investments toward the poorest to reduce child health inequalities.

## Appendix for paper ‘Assessing levels and trends of inequality in child health outcomes and the coverage of child health interventions in developing countries: from 2000 to 2014’

**Table A1.** Data sources of the countries involved in this study, 238 surveys.

Country	Round 1	Round 2	Round 3	Countdown	SSA
Afghanistan			MICS 2010	Yes	No
Albania	MICS 2000	MICS 2005, DHS 2008		No	No
Armenia	DHS 2000	DHS 2005	DHS 2010	No	No
Azerbaijan	MICS 2000	DHS 2006		Yes	No
Bangladesh	DHS 2004	MICS 2006, DHS 2007	DHS 2011, MICS 2012, DHS 2014	Yes	No
Belarus		MICS 2005	MICS 2012	No	No
Belize		MICS 2006	MICS 2011	No	No
Benin	DHS 2001	DHS 2006	DHS 2011	Yes	Yes
Bolivia	MICS 2000, DHS 2003	DHS 2008		Yes	No
Bosnia and Herzegovina		MICS 2006	MICS 2011	No	No
Burkina Faso	DHS 2003	MICS 2006	DHS 2010	Yes	Yes
Burundi	MICS 2000	MICS 2005	DHS 2010	Yes	Yes
Cambodia	DHS 2000	DHS 2005	DHS 2010, DHS 2014	Yes	No
Cameroon	DHS 2004	MICS 2006	DHS 2011	Yes	Yes
Central African Republic	MICS 2000	MICS 2006	MICS 2010	Yes	Yes
Chad	MICS 2000, DHS 2004		MICS 2010	Yes	Yes
Colombia	DHS 2000	DHS 2005	DHS 2010	No	No
Comoros	MICS 2000		DHS 2012	Yes	Yes
Congo, Dem. Rep.	MICS 2001	DHS 2007	MICS 2010, DHS 2013	Yes	Yes
Congo, Rep.		DHS 2005	DHS 2011	No	Yes
Costa Rica			MICS 2011	No	No
Cote d’Ivoire	MICS 2000	MICS 2006	DHS 2011	Yes	Yes
Dominican Republic	DHS 2000, DHS 2002	DHS 2007	DHS 2013	No	No
Egypt	DHS 2000	DHS 2005, DHS 2008	DHS 2014	Yes	No
Ethiopia	DHS 2000	DHS 2005	DHS 2011	Yes	Yes
Gabon	DHS 2000		DHS 2012	Yes	Yes
Gambia	MICS 2000	MICS 2005		Yes	Yes
Georgia		MICS 2005		No	No
Ghana	DHS 2003	MICS 2006, DHS 2008	MICS 2011, DHS 2014	Yes	Yes
Guinea		DHS 2005	DHS 2012	Yes	Yes
Guinea-Bissau	MICS 2000	MICS 2006		Yes	Yes
Guyana	MICS 2000	MICS 2006, DHS 2009		No	No
Haiti	DHS 2000	DHS 2005	DHS 2012	Yes	No
Honduras		DHS 2005	DHS 2011	No	No
India		DHS 2005		Yes	No
Indonesia	DHS 2002	DHS 2007	DHS 2012	Yes	No
Iraq	MICS 2000		MICS 2011	Yes	No
Jordan	DHS 2002	DHS 2007	DHS 2012	No	No
Kazakhstan		MICS 2006	MICS 2010	No	No
Kenya	MICS 2000, DHS 2003	DHS 2008	DHS 2014	Yes	Yes
Kyrgyzstan		MICS 2005	DHS 2012, MICS 2014	Yes	No
Lao PDR	MICS 2000	MICS 2006	MICS 2011	Yes	No
Lesotho	DHS 2004	DHS 2009		Yes	Yes
Liberia		DHS 2007	DHS 2013	Yes	Yes
Macedonia, FYR		MICS 2005	MICS 2011	No	No
Madagascar	MICS 2000, DHS 2003	DHS 2008		Yes	Yes
Malawi	DHS 2000, DHS 2004	MICS 2006	DHS 2010, MICS 2013	Yes	Yes
Maldives		DHS 2009		No	No
Mali	DHS 2001	DHS 2006	DHS 2012	Yes	Yes
Mauritania		MICS 2007		Yes	Yes
Moldova	MICS 2000	DHS 2005	MICS 2012	No	No
Mongolia	MICS 2000	MICS 2005	MICS 2010, MICS 2013	No	No
Montenegro		MICS 2005	MICS 2013	No	No
Morocco	DHS 2003			Yes	No
Mozambique	DHS 2003	MICS 2008	DHS 2011	Yes	Yes
Namibia	DHS 2000	DHS 2006	DHS 2013	No	Yes
Nepal	DHS 2001	DHS 2006	DHS 2011, MICS 2014	Yes	No
Nicaragua	DHS 2001			No	No
Niger	MICS 2000	DHS 2006	DHS 2012	Yes	Yes
Nigeria	DHS 2003	DHS 2008	MICS 2011, DHS 2013	Yes	Yes
Pakistan		DHS 2006	DHS 2012	Yes	No
Peru	DHS 2000, DHS 2004	DHS 2007, DHS 2009	DHS 2010, DHS 2011, DHS 2012	Yes	No
Philippines	DHS 2003	DHS 2008	DHS 2013	Yes	No
Rwanda	DHS 2000	DHS 2005	DHS 2010, DHS 2014	Yes	Yes
Sao Tome and Principe	MICS 2000	DHS 2008	MICS 2014	Yes	Yes
Senegal	MICS 2000	DHS 2005	DHS 2010, DHS 2012, DHS 2014	Yes	Yes
Serbia		MICS 2005	MICS 2010, MICS 2014	No	No
Sierra Leone	MICS 2000	MICS 2005, DHS 2008	MICS 2010, DHS 2013	Yes	Yes
Somalia		MICS 2006		Yes	Yes
State of Palestine			MICS 2014	No	No
Suriname	MICS 2000	MICS 2006	MICS 2010	No	No
Swaziland	MICS 2000	DHS 2006	MICS 2010	Yes	Yes
Syria		MICS 2006		No	No
Tajikistan	MICS 2000	MICS 2005		Yes	No
Tanzania	DHS 2004		DHS 2010	Yes	Yes
Thailand		MICS 2005		No	No
Timor-Leste		DHS 2009		No	No
Togo	MICS 2000	MICS 2006	MICS 2010, DHS 2013	Yes	Yes
Trinidad and Tobago	MICS 2000	MICS 2006		No	No
Turkey	DHS 2003			No	No
Uganda	DHS 2000	DHS 2006	DHS 2011	Yes	Yes
Ukraine		MICS 2005, DHS 2007	MICS 2012	No	No
Uzbekistan		MICS 2006		Yes	No
Vanuatu		MICS 2007		No	No
Vietnam	MICS 2000	MICS 2006	MICS 2010, MICS 2013	Yes	No
Yemen		MICS 2006	DHS 2013	Yes	No
Zambia	MICS 2000, DHS 2001	DHS 2007	DHS 2013	Yes	Yes
Zimbabwe		DHS 2005, MICS 2009	DHS 2010, MICS 2014	Yes	Yes

**Table A2.** Wealth quintile data from WHO Global Health Observatory (GHO) and estimates from our own calculations.

Data from GHO
Afghanistan 2010, Albania 2005, Albania 2008, Armenia 2000, Armenia 2005, Armenia 2010, Azerbaijan 2006, Bangladesh 2004, Bangladesh 2006, Bangladesh 2007, Bangladesh 2011, Belarus 2005, Belarus 2012, Belize 2006, Belize 2011, Benin 2001, Benin 2006, Benin 2011, Bolivia 2003, Bolivia 2008, Bosnia and Herzegovina 2006, Bosnia and Herzegovina 2011, Burkina Faso 2003, Burkina Faso 2006, Burkina Faso 2010, Burundi 2005, Burundi 2010, Cambodia 2000, Cambodia 2005, Cambodia 2010, Cameroon 2004, Cameroon 2006, Cameroon 2011, Central African Republic 2006, Central African Republic 2010, Chad 2004, Colombia 2000, Colombia 2005, Colombia 2010, Comoros 2012, Congo, Dem. Rep. 2007, Congo, Dem. Rep. 2010, Congo, Dem. Rep. 2013, Congo, Rep. 2005, Congo, Rep. 2011, Costa Rica 2011, Cote d’Ivoire 2006, Cote d’Ivoire 2011, Dominican Republic 2000, Dominican Republic 2002, Dominican Republic 2007, Egypt 2000, Egypt 2005, Egypt 2008, Ethiopia 2000, Ethiopia 2005, Ethiopia 2011, Gabon 2000, Gabon 2012, Gambia 2005, Georgia 2005, Ghana 2003, Ghana 2006, Ghana 2008, Ghana 2011, Guinea 2005, Guinea 2012, Guinea-Bissau 2006, Guyana 2006, Guyana 2009, Haiti 2000, Haiti 2005, Haiti 2012, Honduras 2005, Honduras 2011, India 2005, Indonesia 2002, Indonesia 2007, Indonesia 2012, Iraq 2011, Jordan 2002, Jordan 2007, Jordan 2012, Kazakhstan 2006, Kazakhstan 2010, Kenya 2003, Kenya 2008, Kyrgyzstan 2005, Kyrgyzstan 2012, Lao PDR 2006, Lao PDR 2011, Lesotho 2004, Lesotho 2009, Liberia 2007, Liberia 2013, Macedonia, FYR 2005, Macedonia, FYR 2011, Madagascar 2003, Madagascar 2008, Malawi 2000, Malawi 2004, Malawi 2006, Malawi 2010, Maldives 2009, Mali 2001, Mali 2006, Mali 2012, Mauritania 2007, Moldova 2005, Mongolia 2005, Mongolia 2010, Montenegro 2005, Morocco 2003, Mozambique 2003, Mozambique 2008, Mozambique 2011, Namibia 2000, Namibia 2006, Nepal 2001, Nepal 2006, Nepal 2011, Nicaragua 2001, Niger 2006, Niger 2012, Nigeria 2003, Nigeria 2008, Nigeria 2011, Nigeria 2013, Pakistan 2006, Pakistan 2012, Peru 2000, Peru 2009, Peru 2010, Peru 2011, Peru 2012, Philippines 2003, Philippines 2008, Philippines 2013, Rwanda 2000, Rwanda 2005, Rwanda 2010, Sao Tome and Principe 2008, Senegal 2005, Senegal 2010, Senegal 2012, Serbia 2005, Serbia 2010, Sierra Leone 2005, Sierra Leone 2008, Sierra Leone 2010, Sierra Leone 2013, Somalia 2006, Suriname 2006, Suriname 2010, Swaziland 2006, Swaziland 2010, Syria 2006, Tajikistan 2005, Tanzania 2004, Tanzania 2010, Thailand 2005, Timor-Leste 2009, Togo 2006, Togo 2010, Trinidad and Tobago 2006, Turkey 2003, Uganda 2000, Uganda 2006, Uganda 2011, Ukraine 2005, Ukraine 2007, Uzbekistan 2006, Vanuatu 2007, Vietnam 2006, Vietnam 2010, Yemen 2006, Zambia 2001, Zambia 2007, Zimbabwe 2005, Zimbabwe 2009, Zimbabwe 2010
**Estimates from our calculations**
Albania 2000, Azerbaijan 2000, Bangladesh 2012, Bangladesh 2014, Bolivia 2000, Burundi 2000, Cambodia 2014, Central African Republic 2000, Chad 2000, Chad 2010, Comoros 2000, Congo, Dem. Rep. 2001, Cote d’Ivoire 2000, Dominican Republic 2013, Egypt 2014, Gambia 2000, Ghana 2014, Guinea-Bissau 2000, Guyana 2000, Iraq 2000, Kenya 2000, Kenya 2014, Kyrgyzstan 2014, Lao PDR 2000, Madagascar 2000, Malawi 2013, Moldova 2000, Moldova 2012, Mongolia 2000, Mongolia 2013, Montenegro 2013, Namibia 2013, Nepal 2014, Niger 2000, Peru 2004, Peru 2007, Rwanda 2014, Sao Tome and Principe 2000, Sao Tome and Principe 2014, Senegal 2000, Senegal 2014, Serbia 2014, Sierra Leone 2000, State of Palestine 2014, Suriname 2000, Swaziland 2000, Tajikistan 2000, Togo 2000, Togo 2013, Trinidad and Tobago 2000, Ukraine 2012, Vietnam 2000, Vietnam 2013, Yemen 2013, Zambia 2000, Zambia 2013, Zimbabwe 2014

**Table A3.** Definition of child health indicators.

Category	Indicator	Abbreviation	Definition of the indicators
Child health outcomes	Infant mortality	IMR	Infant mortality per 1,000 live births
	Under-five mortality	U5MR	Under-five mortality per 1,000 live births
	Stunting	STU	Proportion of children aged 0–59 months whose height for age is less than 2 standard deviation median height for age of reference population
Child health interventions	Improved sanitation	SAN	Proportion of members of households with improved sanitary facilities
	Improved water supply	WAT	Proportion of members of households with improved drinking water source or using bottled water if source for nondrinking water is improved
	Skilled birth attendant	SBA	Proportion of mothers aged 15–49 who had their delivery assisted by a skilled health professional
	Four or more antenatal care visits	ANC4	Proportion of mothers aged 15–49 who had four or more times of antenatal care visits during last pregnancy (in the two or three years preceding the survey)
	One or more antenatal care visits	ANC1	Proportion of mothers aged 15–49 who had one or more times of antenatal care visits during last pregnancy (in the two or three years preceding the survey)
	Family planning needs satisfied	FPS	Proportion of all women 15–49 using contraception among those who are fecund, in union, and in need of contraception
	Composite coverage index	CCI	Weighted mean of the coverage of eight interventions. The formula is as below: $CCI=\frac{1}{4}*\left(FPS+\frac{SBA+ANCI}{2}+\frac{2DPT3+MSL+BCG}{4}+\frac{ORT+TPN}{2}\right)$
	DPT immunization	DPT3	Proportion of children aged 12–23 months who received at least three doses of DPT vaccine
	Measles immunization	MSL	Proportion of children aged 12–23 months who received a dose of measles vaccine
	BCG immunization	BCG	Proportion of children aged 12–23 months who received a dose of BCG vaccine
	Polio immunization	POI	Proportion of children aged 12–23 months who received at least three doses of polio vaccine
	Full immunization	FULL	Proportion of children aged 12–23 months who received three doses of DPT and Polio vaccines and one dose of BCG and measles vaccines
	Oral rehydration therapy	ORT	Proportion of children aged 0–59 months who received special package of oral-salt-water solution (ORS) or prepackaged ORS liquid
	Care seeking for diarrhea	TDIA	Proportion of children aged 0–59 months who had diarrhea in the past 2 weeks had ORS or sought for care from health facilities, including hospitals, health centers, dispensaries, village health workers, maternal and child health clinics, mobile/outreach clinics, private physicians
	Care seeking for suspected pneumonia	TPN	Proportion of children aged 0–59 months who had suspected pneumonia (cough and rapid breathing, or problem in the chest) in the past 2 weeks sought for care from health facilities, including hospitals, health centers, dispensaries, village health workers, maternal and child health clinics, mobile/outreach clinics, private physicians
	Vitamin A supplementation	VITA	Proportion of children aged 6–59 months who received a vitamin A dose in the past 6 months
	Insecticide-treated bed net for children	ITN	Proportion of children aged 0–59 months who slept under a treated bed net the night before the interview

Child health interventions included in previous studies are highlighted in light purple.

**Table A4.** Comparison of three inequality measurements.

Inequality measurement	Calculation	Features	Limitations
Difference between Q5 and Q1	Absolute inequality measures the different in percentage points between the most advantaged group (Q5) and the least advantaged group (Q1)	Improvement in this measurement implies Q1 group improve faster in terms of the absolute number in the health indicator comparing to the Q5 group, or Q5 group experienced faster absolute health deterioration than the Q1 group	Even if the Q1 group improves at a slower rate than the Q5 group, the absolute difference may still shrink. For example, a country’s infant mortality was 200 per 1,000 live births for the Q1 group and 100 for the Q5 group in 1990. The absolute difference was 200–100 = 100. In the decade, the infant mortality in Q1 group declined by 20%, and the Q5 group declined faster by 30%. The absolute difference decreased to 200 × (1 – 20%) – 100 × (1 – 30%) = 90. In this case, the absolute difference between Q1 and Q5 groups decreases from 100 to 90 per 1,000 live births, which indicates the Q1 and Q5 groups are being more equal. However, in fact, the Q5 group improves at a faster rate than the Q1 group, which appears to suggest the Q5 group did better than the Q1 group, and these two groups should become less equal. This is counterintuitive. It will not reflect the overall status of a health indicator in a country. For example, the absolute inequality of a country with 90% the children in Q1 and 100% of the children in Q5 covered by polio vaccine is the same as that of a country with 5% of the children in Q1 and 15% of the children in Q5 covered. Yet intuitively, the latter one should be of higher concern.
Concentration index	Concentration indexes were generated from the concentration curves. The detailed calculation method could refer to the World Bank instruction^a^	Concentration index quantified the degree of socioeconomic-related inequality in a health variable, which incorporates information from all income groups instead of simply the poorest and the richest	It has higher data requirements than the other equality measurements. There are usually two ways to obtain concentration index. One approach is using grouped data, which requires data on the health indicator and the number of individuals for each income group. The second approach uses micro-data, which requires health status data and wealth score for each individual. This study adopted the first approach. It could be sensitive to the living standards measure, such as consumption, expenditure, and wealth index. We follow Wagstaff’s study and use wealth index to measure living standard.
Ratio of Q5 to Q1	Ratio of Q5 to Q1 measures the rate of the health indicator between the most advantaged group and the least advantaged group	Improvement in the ratio implies a faster relative rate of health improvement among disadvantaged groups	If the ratio is very large/small, we do not have a clear clue of whether it is because the Q1 group is doing particularly poor/good or the Q5 group is doing very well/poor. The ratio will improve if the health status of the Q5 gets worse. For example, if 90% of the children in Q5 received polio vaccine and 10% of the children in Q1 received it, the ratio of Q5 to Q1 is 9. If the polio vaccine coverage of Q5 decreased to 20%, while that of Q1 remained unchanged, the ratio would drop to 2, and we would falsefully conclude that we are making progress toward health equality.

^a^The World Bank instruction can be found from: http://siteresources.worldbank.org/INTPAH/Resources/Publications/459843–1195594469249/HealthEquityCh8.pdf

**Table A5.** Number of countries with available data on the three health outcome and 17 health intervention indicators.

	88 countries with available DHS/MICS surveys after 2000	41 countries with available data in all three survey rounds
*Child health outcome indicators*		
Infant mortality	Available in 52 countries	21 countries are with valid data in all three rounds
Under-five mortality	Available in 52 countries	21 countries are with valid data in all three rounds
Stunting	Available in 80 countries	34 countries are with valid data in all three rounds
*Child health intervention indicators*		
Improved sanitation	Available in 83 countries	40 countries are with valid data in all three rounds
Improved water supply	Available in 83 countries	39 countries are with valid data in all three rounds
Skilled birth attendant	Available in 88 countries	39 countries are with valid data in all three rounds
Four or more antenatal care visits	Available in 64 countries	21 countries are with valid data in all three rounds
One or more antenatal care visits	Available in 88 countries	39 countries are with valid data in all three rounds
Family planning needs satisfied	Available in 61 countries	22 countries are with valid data in all three rounds
Composite coverage index	Available in 38 countries	9 countries are with valid data in all three rounds
DPT immunization	Available in 87 countries	38 countries are with valid data in all three rounds
Measles immunization	Available in 87 countries	39 countries are with valid data in all three rounds
BCG immunization	Available in 85 countries	38 countries are with valid data in all three rounds
Polio immunization	Available in 87 countries	38 countries are with valid data in all three rounds
Full immunization	Available in 84 countries	37 countries are with valid data in all three rounds
Oral rehydration therapy	Available in 85 countries	38 countries are with valid data in all three rounds
Care seeking for diarrhea	Available in 86 countries	40 countries are with valid data in all three rounds
Care seeking for suspected pneumonia	Available in 67 countries	31 countries are with valid data in all three rounds
Vitamin A supplementation	Available in 64 countries	29 countries are with valid data in all three rounds
Insecticide-treated bed net for children	Available in 38 countries	15 countries are with valid data in all three rounds

The full names and the definitions of the indicators are in appendix Table 1. The data for Tajikistan 2012 cannot be downloaded from Demographic Health Survey dataset, so we did not include it in our analysis. If an indicator from a survey is with partially complete data (for example, the data for Q1group are complete, yet with too many missing values for Q5 group to generate valid quintile value), we count such indicators as ‘Not available’.

**Table A6.** Summary of three child health outcomes and 17 child health interventions with 95% CI, most recent survey data for 88 countries.

					Difference in Q5 – Q1			
	No. of countries	Overall value	Q1	Q5	Mean	IQR	Bottom decile (10%)	Top decile (10%)
Child health outcomes								
Infant mortality per 1,000 live births	52	50.4 [44.8, 56.0]	58.9 [52.6, 65.3]	36.3 [31.3, 41.4]	−22.6 [−26.8, −18.3]	19.2	−45.2	−2.0
Under-five mortality per 1,000 live births	52	76.0 [65.6, 86.4]	91.8 [79.8, 103.8]	49.5 [41.5, 57.5]	−42.3 [−49.5, −35.1]	31.0	−77.3	−16.4
Stunting prevalence (%)	80	28.0% [25.1, 30.8]	35.4% [32.1, 38.7]	17.2% [14.8, 19.5]	−18.2% [−20.5, −15.9]	13.8%	−34.5%	−4.8%
**Child health interventions**								
Access to improved sanitation (%)	83	59.5% [53.0, 65.9]	38.2% [30.3, 46.2]	87.8% [83.6, 91.9]	49.5% [42.7, 56.2]	62.6%	5.3%	89.1%
Access to improved water (%)	83	75.5% [71.9, 79.1]	58.9% [53.5, 64.3]	93.0% [91.3, 94.7]	34.1% [29.5, 38.6]	35.6%	5.6%	62.5%
Skilled birth attendant (%)	88	74.0% [69.2, 78.8]	58.8% [52.3, 65.4]	92.9% [91.0, 94.9]	34.1% [28.8, 39.4]	44.7%	0.5%	68.6%
Four or more antenatal care visits (%)	64	61.1% [55.7, 66.5]	49.7% [43.2, 56.1]	77.7% [73.5, 81.8]	27.9% [23.3, 32.6]	28.1%	3.2%	53.6%
One or more antenatal care visits (%)	88	87.2% [84.1, 90.3]	78.8% [74.2, 83.5]	95.7% [94.3, 97.1]	16.9% [13.0, 20.7]	25.2%	0.4%	43.6%
Family planning need satisfied (%)	61	59.3% [54.2, 64.5]	50.5% [44.5, 56.6]	70.8% [66.7, 74.9]	20.2% [16.5, 23.9]	21.1%	4.8%	41.5%
Composite coverage index (%)	38	63.9% [60.0, 67.9]	55.0% [50.1, 59.9]	76.4% [74.0, 78.8]	22.9% [19.3, 26.6]	16.3%	9.3%	35.5%
DPT immunization (%)	87	75.5% [71.7, 79.3]	68.3% [63.4, 73.2]	82.2% [79.1, 85.4]	13.9% [10.2, 17.6]	22.9%	−3.1%	39.1%
Measles immunization (%)	87	77.5% [74.4, 80.6]	70.8% [66.7, 75.0]	84.5% [81.9, 87.0]	13.6% [10.4, 16.8]	21.1%	−2.4%	34.4%
BCG immunization (%)	85	89.0% [86.3, 91.6]	83.3% [79.5, 87.0]	94.3% [92.6, 96.1]	11.0% [8.1, 14.0]	18.3%	−0.9%	29.4%
Polio immunization (%)	87	75.1% [71.6, 78.6]	69.7% [65.5, 73.9]	78.7% [75.4, 82.0]	9.03% [6.3, 11.7]	18.7%	−5.7%	25.9%
Full immunization (%)	84	62.4% [57.8, 67.0]	55.5% [50.0, 60.9]	68.5% [64.2, 72.8]	13.0% [9.3, 16.7]	25.0%	−8.0%	33.8%
ORT for diarrhea (%)	85	40.4% [36.5, 44.4]	37.0% [32.9, 41.1]	44.6% [40.8, 48.3]	8.0% [5.2, 10.8]	16.7%	−5.5%	24.6%
Care seeking for diarrhea (%)	86	56.8% [53.4, 60.2]	52.2% [48.2, 56.2]	62.4% [58.9, 66.0]	10.2% [6.6, 13.8]	18.8%	−6.6%	30.1%
Care seeking for suspected pneumonia (%)	87	59.5% [54.6, 64.3]	52.4% [47.2, 57.6]	70.3% [66.1, 74.6]	18.8% [15.4, 22.2]	22.4%	3.0%	36.5%
Vitamin A supplement (%)	64	53.8% [48.5, 59.1]	49.6% [44.2, 55.0]	58.0% [52.6, 63.5]	8.4% [5.1, 11.7]	17.7%	−8.5%	23.5%
ITN for children (%)	38	38.4% [32.3, 44.6]	36.1% [29.9, 42.2]	37.0% [30.2, 43.9]	1.0% [−3.9, 5.9]	17.1%	−16.9%	22.1%

**Table A6.** Summary of three child health outcomes and 17 child health interventions with 95% CI, most recent survey data for 88 countries (continued).

		Concentration index				Ratio of Q5 to Q1			
	No. of countries	Mean	IQR	Bottom decile (10%)	Top decile (10%)	Mean	IQR	Bottom decile (10%)	Top decile (10%)
	**Child health outcomes**								
Infant mortality per 1,000 live births	52	−8.7 [−10.5, −6.9]	7.8	−17.3	−0.9	0.63 [0.55, 0.70]	0.31	0.33	0.95
Under-five mortality per 1,000 live births	52	−11.0 [−12.7, −9.3]	8.4	−19.6	−3.9	0.54 [0.48, 0.59]	0.27	0.30	0.78
Stunting prevalence (%)	80	−0.14 [−0.17, −0.12]	0.14	−0.27	−0.04	0.49 [0.43, 0.54]	0.33	0.23	0.80
	**Child health interventions**								
Access to improved sanitation (%)	83	26.3 [21.5, 31.0]	35.5	1.0	60.6	35.5 [9.0, 62.2]	12.6	1.1	54.8
Access to improved water (%)	83	11.4 [9.27, 13.6]	14.6	1.6	25.1	2.9 [1.4, 4.5]	1.1	1.1	3.7
Skilled birth attendant (%)	88	13.0 [10.2, 15.8]	18.7	0.1	29.5	2.6 [2.0, 3.1]	1.8	1.0	5.2
Four or more antenatal care visits (%)	64	14.9 [12.1, 17.8]	12.1	1.5	31.5	2.2 [1.8, 2.6]	1.1	1.1	5.4
One or more antenatal care visits (%)	88	6.3 [4.9, 7.8]	7.0	0.1	15.7	1.4 [1.2, 1.5]	0.4	1.0	2.1
Family planning need satisfied (%)	61	8.6 [6.6, 10.6]	9.5	1.2	19.8	1.8 [1.4, 2.1]	0.7	1.1	2.7
Composite coverage index (%)	38	7.8 [6.1, 9.5]	6.3	2.5	14.0	1.5 [1.3, 1.8]	0.5	1.1	2.1
DPT immunization (%)	87	5.5 [3.8, 7.1]	8.2	−0.6	16.0	1.4 [1.2, 1.7]	0.4	1.0	2.1
Measles immunization (%)	87	4.5 [3.3, 5.6]	5.8	−1.6	10.1	1.3 [1.1, 1.4]	0.4	1.0	1.8
BCG immunization (%)	85	3.0 [1.9, 4.1]	3.6	−0.4	7.2	1.2 [1.0, 1.3]	0.2	1.0	1.4
Polio immunization (%)	87	2.2 [0.4, 4.0]	5.4	−1.6	8.1	1.1 [1.1, 1.2]	0.3	0.9	1.5
Full immunization (%)	84	7.0 [4.7, 9.3]	9.9	−2.2	17.8	1.6 [1.2, 1.9]	0.7	0.9	2.4
ORT for diarrhea (%)	85	4.2 [1.9, 6.5]	10.4	−7.1	18.7	1.5 [1.2, 1.7]	1.5	0.8	2.5
Care seeking for diarrhea (%)	86	2.7 [1.1, 4.4]	6.0	−4.2	10.5	1.4 [1.1, 1.6]	0.4	0.9	2.1
Care seeking for suspected pneumonia (%)	87	4.2 [2.8, 5.6]	6.6	−2.9	13.4	1.6 [1.4, 1.9]	0.8	1.0	2.6
Vitamin A supplement (%)	64	3.8 [1.4, 6.1]	6.5	−2.9	9.9	1.3 [1.1, 1.4]	0.4	0.8	1.8
ITN for children (%)	38	0.7 [−3.0, 4.5]	11.7	−12.7	17.1	1.2 [0.9, 1.5]	0.6	0.5	2.1

**Table A7.** Summary of three child health outcomes and 17 child health interventions with 95% CI, most recent survey data for 88 countries, weighted by population size.

					Difference in Q5 – Q1			
	No. of countries	Overall value	Q1	Q5	Mean	IQR	Bottom decile (10%)	Top decile (10%)
**Child health outcomes**								
Infant mortality per 1,000 live births	52	55.8 [51.3, 60.3]	71.5 [66.3, 76.7]	34.4 [30.7, 38.0]	−37.1 [−41.0, −33.2]	21.1	−47.8 [−51.7, −43.8]	−13.8 [−17.7, −9.9]
Under-five mortality per 1,000 live births	52	77.3 [69.2, 85.4]	106. [96.4, 116.]	43.4 [37.7, 49.1]	−62.7 [−69.7, −55.8]	34.0	−77.3 [−84.2, −70.3]	−23.0 [−29.9, −16.0]
Stunting prevalence (%)	80	38.1% [35.7, 40.6]	50.2% [47.2, 53.3]	21.6% [19.9, 23.4]	−28.5% [−30.7, −26.4]	13.8%	−35.7% [−37.8, −33.5]	−12.9% [−15.0, −10.7]
**Child health interventions**								
Access to improved sanitation (%)	83	52.6% [47.2, 58.1]	24.7% [18.0, 31.4]	86.7% [83.4, 89.9]	61.9% [56.2, 67.7]	60.1%	16.6% [10.8, 22.3]	82.7% [77.0, 88.5]
Access to improved water (%)	83	79.1% [75.6, 82.6]	65.4% [60.4, 70.5]	91.9% [90.3, 93.5]	26.4% [22.3, 30.5]	33.9%	10.8% [6.78, 14.9]	55.4% [51.3, 59.5]
Skilled birth attendant (%)	88	62.1% [57.9, 66.3]	37.2% [31.5, 42.8]	90.1% [88.1, 92.1]	52.9% [48.4, 57.5]	42.7%	17.8% [13.2, 22.3]	68.8% [64.2, 73.3]
Four or more antenatal care visits (%)	64	49.1% [44.4, 53.9]	27.6% [21.6, 33.6]	76.8% [73.3, 80.2]	49.1% [44.2, 54.0]	30.1%	21.6% [16.7, 26.5]	65.8% [60.8, 70.7]
One or more antenatal care visits (%)	88	80.6% [77.5, 83.6]	63.1% [58.4, 67.9]	96.0% [94.8, 97.1]	32.8% [28.7, 36.9]	27.2%	5.6% [1.5, 9.7]	52.1% [47.9, 56.2]
Family planning need satisfied (%)	61	71.8% [67.3, 76.4]	61.2% [55.6, 66.8]	82.1% [79.2, 85.0]	20.8% [16.9, 24.7]	22.1%	3.1% [−0.8, 7.0]	42.7% [38.7, 46.6]
Composite coverage index (%)	38	65.1% [61.5, 68.7]	49.9% [45.6, 54.2]	80.6% [79.0, 82.2]	30.7% [27.0, 34.4]	16.6%	14.8% [11.0, 18.5]	40.7% [36.9, 44.4]
DPT immunization (%)	87	66.6% [63.0, 70.1]	48.6% [43.3, 53.9]	83.7% [81.7, 85.8]	35.1% [30.7, 39.5]	23.1%	5.2% [0.8, 9.7]	58.1% [53.6, 62.5]
Measles immunization (%)	87	69.6% [66.6, 72.6]	53.6% [49.1, 58.1]	85.5% [83.9, 87.2]	31.9% [28.1, 35.8]	22.8%	6.4% [2.6, 10.2]	47.7% [43.8, 51.5]
BCG immunization (%)	85	84.1% [81.5, 86.7]	71.8% [67.6, 76.0]	95.4% [94.3, 96.4]	23.5% [19.7, 27.3]	20.1%	1.8% [−1.9, 5.6]	31.6% [27.8, 35.3]
Polio immunization (%)	87	77.0% [74.4, 79.7]	68.3% [65.3, 71.3]	84.5% [82.5, 86.6]	16.2% [14.3, 18.1]	17.8%	2.4% [0.5, 4.3]	25.9% [24.0, 27.7]
Full immunization (%)	84	55.0% [51.1, 58.9]	38.5% [33.4, 43.7]	72.1% [69.4, 74.7]	33.5% [29.5, 37.4]	20.9%	3.3% [−0.6, 7.2]	52.0% [48.0, 55.9]
ORT for diarrhea (%)	85	37.8% [34.2, 41.4]	31.1% [27.1, 35.1]	46.3% [43.4, 49.3]	15.2% [12.2, 18.1]	16.3%	−5.0% [−7.9, −2.0]	24.5% [21.5, 27.4]
Care seeking for diarrhea (%)	86	68.3% [65.3, 71.2]	59.6% [56.4, 62.8]	76.4% [73.5, 79.3]	16.8% [14.3, 19.3]	18.8%	3.3% [0.8, 5.8]	24.4% [21.9, 26.9]
Care seeking for suspected pneumonia (%)	87	64.3% [59.8, 68.9]	55.1% [50.5, 59.6]	73.6% [70.1, 77.0]	18.4% [15.7, 21.1]	22.3%	6.6% [3.9, 9.3]	36.4% [33.6, 39.1]
Vitamin A supplement (%)	64	40.2% [33.7, 46.6]	34.7% [28.7, 40.8]	45.2% [38.6, 51.7]	10.4% [7.51, 13.3]	17.9%	2.4% [−0.5, 5.3]	24.5% [21.5, 27.4]
ITN for children (%)	38	37.3% [30.5, 44.0]	33.4% [26.9, 39.9]	38.2% [31.5, 44.8]	4.8% [1.3, 8.3]	16.1%	−2.2% [−5.6, 1.3]	22.1% [18.6, 25.5]

We took two steps to generate the mean estimate and the 95% confidence intervals for each indicator. First, for each country with available data, we calculated the point estimates of all indexes (overall value, Q1, Q5, difference between Q5 and Q1, concentration index, and ratio of Q5 to Q1) following the method of O’Donnell et al. (O’Donnell O, Wagstaff A. Analyzing health equity using household survey data: a guide to techniques and their implementation. World Bank Publications, 2007) Second, we treated each country as a subject to calculate the mean values and weighted them according to their population size. Then we generated standard errors for the point estimates and calculated 95% CI based on the point estimate and the standard errors.

**Table A8.** Absolute difference and the 95% CI between Q5 and Q1 in the three child health outcomes by survey round and country, 41 countries.

	Infant mortality per 1,000 live births			Under-five mortality per 1,000 live births			Stunting prevalence (%)		
Country	Round 1	Round 2	Round 3	Round 1	Round 2	Round 3	Round 1	Round 2	Round 3
Armenia	−26.8 [−43.5, −10.0]	−26.7 [−41.6, −11.7]	−.3 [−16.2, 15.7]	−32.9 [−51.0, −14.7]	−27.8 [−46.1, −9.45]	−3.9 [−21.3, 13.50]	−9.5 [−15.7, −3.27]	−1.6 [−11.5, 8.302]	−7.4 [−15.9, 1.2]
Bangladesh^a^	−25.5 [−40.0, −10.9]	−30.3 [−45.4, −15.1]	−19 [−34, −4]	−49.3 [−66.0, −32.5]	−42.1 [−58.7, −25.4]	−23.0 [−40.8, −5.2]	−31.6 [−36.3, −26.8]	−27.4 [−32.5, −22.2]	−29.7 [−35.2, −24.1]
Benin^a^^b^	−62.3 [−81.5, −43.0]	−27.7 [−39.2, −16.1]	−22.1 [−31.8, −12.3]	−105.2 [−131.0, −79.4]	−67.9 [−83.4, −52.3]	−43.0 [−55.8, −30.1]	−19.1 [−24.1, −14.0]	−20.9 [−24.2, −17.5]	−11.5 [−16.3, −6.7]
Burkina Faso^a^^b^	−19.4 [−39.0, 0.3]	−36.1 [−48.1, −24.0]	−61.1 [−89.1, −33.0]	−78.8 [−97.4, −60.1]	−25 [−29.9, −20.0]	−25 [−31.6, −18.3]	−24 [−28.1, −19.8]		
Burundi^a^^b^			−44.4 [−60.5, −28.2]		−67.8 [−88.0, −47.5]	−16.1 [−21.0, −11.1]	−28.1 [−33.9, −22.2]		
Cambodia^a^	−59.9 [−75.5, −44.2]	−65.5 [−78.9, −52.0]	−46 [−60.7, −31.2]	−92.2 [−109.3, −75.1]	−83 [−98.0, −67.9]	−57 [−73.2, −40.7]	−26.3 [−32.5, −20.0]	−29.4 [−34.9, −23.8]	−23.4 [−28.3, −18.4]
Cameroon^a^^b^	−49.1 [−65.9, −32.2]	−38.8 [−52.4, −25.1]	−102.9 [−125.0, −78.8]	−111.0 [−129.0, −93.0]	−29.6 [−35.2, −23.9]	−34.4 [−39.1, −29.6]	−36.4 [−40.7, −32.0]		
Central African Republic^a^^b^						−7.60 [−10.5, −4.61]	−17.8 [−23.3, −12.2]	−15 [−19.4, −10.5]	
Colombia	−14.7 [−24.0, −5.4]	−17.5 [−24.9, −10.0]	−9.6 [−14.6, −4.50]	−19.1 [−29.6, −8.55]	−23.3 [−31.2, −15.3]	−16.4 [−21.9, −10.8]	−17.6 [−21.4, −13.7]	−20.5 [−22.8, −18.1]	−12.9 [−15.3, −10.4]
Congo, Dem. Rep.^a^^b^	−54.9 [−83.2, −26.5]	−13.8 [−25.9, −1.69]	−86.1 [−117.0, −54.7]	−39.8 [−56.4, −23.1]	**−18.5 [−21.1, −15.9]**	**−21 [−29.8, −12.1]**	**−26.8 [−31.6, −21.9]**		
Cote d’Ivoire^a^^b^			−23.3 [−44.1, −2.43]		−40.6 [−64.8, −16.3]	−20.3 [−25.8, −14.7]	−24.8 [−30.6, −18.9]		
Dominican Republic	−29.8 [−39.7, −19.8]	−17.8 [−29.2, −6.36]	−24 [−37.6, −10.3]	−42.2 [−53.5, −30.8]	−25.8 [−37.9, −13.6]	−25 [−39.2, −10.7]	−15.9 [−18.6, −13.1]	−12 [−14.6, −9.35]	−7.4 [−11.1, −3.69]
Egypt^a^	−46.4 [−56.4, −36.3]	−25.1 [−32.6, −17.5]	−18 [−26.4, −9.51]	−64.5 [−75.3, −53.6]	−29.5 [−37.3, −21.6]	−23 [−31.7, −14.2]	−16.3 [−19.8, −12.7]	−2.49 [−6.08, 1.080]	−.700 [−3.21, 1.816]
Ethiopia^a^^b^	**1.8 [−15.2, 18.9]**	**−19.1 [−33.8**, **−4**.**31]**	**−31.2 [−45.9**, **−1****6.4]**	−11.7 [−36.6, 13.2]	−37.5 [−56.8, −18.1]	−52.1 [−72.0, −32.1]	−11.6 [−17.5, −5.67]	−12.5 [−19.7, −5.20]	−19.7 [−24.3, −15.0]
Ghana^a^^b^	−4.3 [−22.7, 14.2]	−13.4 [−32.6, 5.9]	−4 [−26.2, 18.3]	−40.6 [−68.0, −13.1]	−42.6 [−65.8, −19.3]	−28 [−54.3, −1.60]	−29.6 [−35.1, −24.0]	−19.1 [−24.7, −13.4]	−16.3 [−21.6, −10.9]
Haiti^a^	−.9 [−30.9, 29.11]	−33.9 [−51.8, −15.9]	−11.4 [−25.5, 2.8]	−54.4 [−82.7, −26.0]	−68.9 [−93.8, −43.9]	−42.3 [−60.5, −24.0]	−27.3 [−32.1, −22.4]	−32.4 [−38.0, −26.7]	−24 [−28.4, −19.5]
Indonesia^a^	−43.4 [−52.7, −34.0]	−29.9 [−39.2, −20.5]	−35.1 [−42.0, −28.1]	−54.8 [−65.3, −44.2]	−46.7 [−57.2, −36.1]	−47.9 [−56.4, −39.3]			
Jordan	−10.3 [−20.2, –.337]	0 [−13.8, 13.8]	−17.1 [−27.9, −6.3]	−11.8 [−22.8, −0.8]	−3.0 [−17.6, 11.6]	−16.4 [−27.8, −4.9]	−10.9 [−14.0, −7.71]	−9 [−13.4, −4.54]	−12.1 [−15.5, −8.65]
Kenya^a^^b^	−34.1 [−54.3, −13.8]	−8.3 [−27.2, 10.7]	−2.0 [−21.5, 17.6]	−56.7 [−85.1, −28.2]	−27.9 [−50.7, −5.05]	−10 [−35.2, 15.23]	−19.3 [−24.2, −14.3]	−19 [−24.4, −13.5]	−22.1 [−24.7, −19.4]
Lao PDR^a^							**−12.7 [−19.1**, **−6**.**37]**	**−34.8 [−40.4**, **−2****9.1]**	**−40.9 [−44.3**, **−3****7.4]**
Malawi^a^^b^	−42.4 [−59.3, −25.4]		−71.4 [−94.3, −48.4]		−21.8 [−26.6, −16.9]	−13.5 [−17.3, −9.61]	−14.1 [−17.1, −11.1]		
Mali^a^^b^	−47.8 [−65.7, −29.8]	−43.8 [−57.6, −29.9]	−17.7 [−31.0, −4.4]	−100.0 [−123.4, −76.6]	−110 [−131.5, −88.5]	−51.4 [−69.4, −33.3]	−25.4 [−30.3, −20.4]	−22.4 [−26.2, −18.5]	−25.4 [−30.2, −20.5]
Moldova		−4.2 [−15.8, 7.4]			−10.7 [−23.4, 2.031]		−8.0 [−12.9, −3.1]	−7.40 [−11.5, −3.23]	
Mongolia							−19.4 [−22.2, −16.6]	−12.5 [−17.3, −7.60]	−9.90 [−11.8, −7.93]
Mozambique^a^^b^	−71.7 [−90.3, −53.0]	−18.9 [−33.2, −4.6]	−88.7 [−110.0, −66.6]	−39.2 [−58.2, −20.1]	−28.7 [−33.1, −24.2]	−25 [−29.3, −20.6]	−26.6 [−30.5, −22.6]		
Namibia^b^	−19.8 [−36.6, −2.95]	−35.7 [−50.3, −21.0]	−29.0 [−46.0, −11.9]	−30.4 [−50.6, −10.1]	−60.8 [−77.7, −43.8]	−36 [−54.9, −17.0]	−20.7 [−28.6, −12.7]	−25.4 [−31.0, −19.7]	−22.6 [−30.6, −14.5]
Nepal^a^	−32.3 [−47.9, −16.6]	−31.8 [−49.0, −14.5]	−62.9 [−82.9, −42.9]	−50.5 [−69.5, −31.4]	−25.5 [−30.4, −20.5]	−31.0 [−36.4, −25.5]	−30.8 [−33.9, −27.7]		
Niger^a^^b^		−23.3 [−38.7, −7.82]	−11.9 [−24.5, 0.8]	−49.5 [−77.7, −21.2]	−32 [−52.0, −11.9]	−9.5 [−13.6, −5.4]	−17.4 [−24.1, −10.6]	−11.6 [−17.4, −5.8]	
Nigeria^a^^b^	−80.0 [−101.2, −58.8]	−41.6 [−51.5, −31.6]	−43.4 [−53.0, −33.7]	−174.0 [−199.0, −148.9]	−129.2 [−144.4, −114.0]	−115.2 [−131.0, −99.4]	−33.0 [−39.2, −26.7]	−28.3 [−31.2, −25.3]	−35.7 [−38.9, −32.4]
Peru^a^	−40 [−47.8, −32.1]	−26.4 [−33.8, −18.9]	−13.2 [−21.5, −4.87]	−51 [−61.4, −40.5]	−30.6 [−39.3, −21.8]	−21.6 [−30.2, −12.9]	−49.5 [−54.9, −44.0]	−41.6 [−45.2, −37.9]	−35 [−38.1, −31.8]
Philippines^a^	−22.1 [−31.6, −12.5]	−25.4 [−34.6, −16.1]	−23.7 [−31.1, −16.2]	−44.8 [−56.3, −33.2]	−41.5 [−52.4, −30.5]	−36.6 [−45.7, −27.4]			
Rwanda^a^^b^	−51.1 [−73.1, −29.0]	−41 [−57.1, −24.8]	−25 [−42.2, −7.74]	−92.8 [−123.3, −62.3]	−90.1 [−113.1, −67.1]	−44 [−67.9, −20.0]	−21.6 [−26.3, −16.8]	−25.2 [−30.8, −19.5]	−27.7 [−32.6, −22.7]
Sao Tome and Principe^a^^b^	−24.8 [−46.9, −2.65]		−57.9 [−88.5, −27.2]	−15.3 [−20.9, −9.84]	−18.2 [−27.5, −8.84]	−12.2 [−16.5, −7.89]			
Senegal^a^^b^		−48.4 [−61.8, −34.9]	−29 [−43.9, −14.0]		−116.6 [−136.1, −97.1]	−64 [−84.6, −43.3]	−18.5 [−21.4, −15.5]	−22 [−27.3, −16.6]	−20.4 [−24.1, −16.6]
Sierra Leone^a^^b^		−56.2 [−84.6, −27.7]	−14.5 [−33.2, 4.2]	−67.3 [−102.6, −32.0]	−42.1 [−66.7, −17.4]	−10.5 [−15.3, −5.81]	−12.8 [−20.2, −5.37]	−14.2 [−19.9, −8.41]	
Suriname							−15.5 [−20.1, −11.0]	−12.4 [−16.6, −8.10]	−7.90 [−11.3, −4.49]
Swaziland^a^^b^		−1 [−28.7, 26.72]			−18 [−48.3, 12.38]		−21.1 [−25.6, −16.6]	−19.6 [−25.8, −13.3]	−27.9 [−33.5, −22.2]
Togo^a^^b^			−29 [−43.9, −14.0]			−74 [−94.6, −53.3]		−18.1 [−23.6, −12.5]	−22.8 [−27.0, −18.5]
Uganda^a^^b^	−3.6 [−21.4, 14.24]	−36.2 [−51.7, −20.6]	−28 [−44.7, −11.2]	−11.7 [−39.4, 16.00]	−53.6 [−74.8, −32.3]	−52.9 [−74.0, −31.7]	−2.59 [−8.54, 3.340]	−18.4 [−25.0, −11.7]	−14.2 [−21.6, −6.76]
Vietnam^a^							−27.2 [−33.0, −21.3]		
Zambia^a^^b^	−58.7 [−77.5, −39.8]	6.1 [−8.90, 21.10]	−16 [−31.8, –.191]	−100.0 [−124.1, −75.9]	−15.4 [−36.1, 5.361]	−42.0 [−63.7, −20.2]	−25.2 [−30.4, −19.9]	−14.2 [−20.9, −7.47]	−18.9 [−21.9, −15.8]

^a^Countdown country; ^b^Sub-Saharan African country.

Countries with significantly enlarged absolute difference between Q1 and Q5 in round 3 comparing to round 1 are shown in bold.

**Table A9.** Absolute difference between Q5 and Q1 in the nine selected child health interventions by survey round and country, 41 countries.

	Access to improved sanitation (%)			Delivered with SBA (%)			Received full immunization (%)		
Country	Round 1	Round 2	Round 3	Round 1	Round 2	Round 3	Round 1	Round 2	Round 3
Armenia	**−0.78 [−3**.**0, 1.43]**	**22.6 [16.1, 29.0]**	**74.2 [68.3, 80.1]**	5.5 [1.3, 9.7]	2.40 [−1.8, 6.7]	0 [0, 0]		1.4 [−20.2, 23.0]	1.9 [−23.9, 27.7]
Bangladesh^a^	65.1 [62.2, 68.0]	57.9 [53.8, 62.0]	51.4 [47.5, 55.3]	**42.1 [37.6, 46.5]**	**49.6 [44.1, 55.0]**	**57.6 [52.3, 62.8]**	29.3 [18.3, 40.2]	8.5 [–.44, 17.4]	21.3 [13.1, 29.4]
Benin^a^^b^	89.8 [87.7, 92.0]	77.8 [75.9, 79.7]	86.8 [84.7, 88.8]	47.3 [40.1, 54.4]	39.0 [34.2, 43.9]	33.1 [28.2, 37.9]	23.4 [11.8, 35]	31.1 [24.8, 37.3]	21 [13.1, 28.8]
Burkina Faso^a^^b^	90.8 [89.1, 92.5]	4.19 [2.89, 5.48]	87.8 [85.5, 90.0]	52.5 [45.9, 59.2]	8.60 [–.53, 17.7]	44.9 [39.6, 50.1]	27.1 [17.4, 36.7]	16.6 [.311, 32.8]	10.6 [2.96, 18.2]
Burundi^a^^b^	**21.2 [18.4, 23.9]**	**4.81 [1.26, 8.37]**	**50.4 [45.6, 55.3]**	30.3 [9.81, 50.9]	29.9 [23.4, 36.3]	27.9 [22.4, 33.3]	17.2 [4.46, 30.0]	5.5 [−4.8, 15.8]	5.70 [−1.7, 13.1]
Cambodia^a^	**82.5 [79.9, 85.1]**	**81.9 [79.2, 84.5]**	**89.7 [87.4, 92.1]**	67.3 [61.2, 73.3]	70 [65.1, 74.8]	21.5 [–.48, 43.6]	39.0 [27.8, 50.3]	20.3 [10.7, 29.8]	29.6 [20.0, 39.1]
Cameroon^a^^b^	79.4 [76.9, 81.9]	63.3 [60.2, 66.4]	73.2 [69.7, 76.7]	**64.7 [58.3, 71.0]**	**76.5 [70.2, 82.7]**	**78.7 [75.1, 82.2]**	24.2 [14.3, 34.0]	26.5 [15.6, 37.3]	37.4 [28.9, 45.8]
Central African Republic^a^^b^	**7.26 [6.23, 8.29]**	**15.2 [13.1, 17.3]**	**16.6 [14.2, 18.9]**	52.8 [47.7, 57.9]	61.9 [55.5, 68.2]	54.1 [47.3, 60.8]	36.6 [29.7, 43.6]	36.8 [26.9, 46.6]	29 [20.7, 37.2]
Colombia	44.9 [41.8, 47.9]	43.1 [41.3, 45.0]	32.7 [31.1, 34.4]	33 [27.8, 38.1]	25.2 [21.8, 28.5]	14.2 [11.7, 16.7]	14.2 [2.31, 26.0]	23.9 [14.9, 32.8]	3.09 [−4.9, 11.1]
Congo, Dem. Rep.^a^^b^	40.8 [39.0, 42.6]	32.8 [26.5, 39.1]	37.4 [33.0, 41.9]	45.7 [41.5, 49.9]	36.3 [30.0, 42.7]	31 [25.9, 36.0]	36.7 [32.0, 41.4]	30.5 [22.0, 38.9]	28.8 [20.9, 36.6]
Cote d’Ivoire^a^^b^	97.8 [97.4, 98.1]	69.7 [67.0, 72.4]	81.8 [77.2, 86.4]	55.6 [52.0, 59.2]	66.1 [59.6, 72.5]	54.7 [49.0, 60.3]	32.7 [28.9, 36.5]	34.4 [23.0, 45.7]	28.8 [17.9, 39.6]
Dominican Republic	44.0 [41.1, 46.8]	43.7 [40.9, 46.5]	21.2 [17.4, 25.0]	5.29 [3.24, 7.35]	4.09 [1.67, 6.52]	.742 [−1.8, 3.37]	9.5 [.194, 18.8]	26.5 [15.9, 37.0]	16.6 [−9.3, 42.5]
Egypt^a^	26.3 [24.1, 28.5]	1.60 [.208, 2.99]	12.0 [10.2, 13.7]	59.7 [55.7, 63.6]	39.1 [35.2, 43.1]	16.5 [14.9, 18.2]	.800 [−4.3, 5.96]	4.79 [.203, 9.39]	6.4 [−82.1, 94.9]
Ethiopia^a^^b^	59.4 [56.2, 62.6]	28.9 [26.2, 31.7]	36.4 [32.7, 40.1]	**24.2 [19.2, 29.1]**	**27.8 [22.3, 33.2]**	**47.5 [40.5, 54.4]**	26.6 [17.9, 35.2]	21.5 [13.1, 29.8]	33.8 [23.3, 44.2]
Ghana^a^^b^	74.7 [70.6, 78.9]	73.8 [69.5, 78.1]	71.4 [66.1, 76.6]	70.2 [63.8, 76.5]	72.7 [66.6, 78.7]	49.7 [42.9, 56.5]	24.7 [11.4, 37.9]	8.09 [−5.2, 21.4]	−1.5 [−15.4, 12.4]
Haiti^a^	**17.2 [12.3, 22.0]**	**64.3 [59.9, 68.7]**	**87.4 [84.5, 90.4]**	67.4 [61.7, 73.0]	62.6 [55.3, 69.8]	71.6 [66.6, 76.5]	16.9 [–.61, 34.4]	22 [8.91, 35.0]	−0.3 [−12.7, 12.1]
Indonesia^a^	54.9 [52.4, 57.3]	59.3 [57.2, 61.5]	45.8 [43.7, 47.8]	52.9 [47.8, 57.9]	49.5 [45.1, 53.8]	37 [33.1, 40.8]	27.5 [18, 37.0]	35.2 [27.0, 43.3]	32.8 [26.2, 39.3]
Jordan	37.3 [34.5, 40.0]	1.59 [1.00, 2.17]	.038 [–.01, .093]	1.40 [–.89, 3.7]	1.59 [–.10, 3.3]	1.40 [–.99, 3.8]	4.80 [−7.8, 17.4]	7.19 [−3.1, 17.5]	1.4 [−16.5, 19.3]
Kenya^a^^b^	**52.9 [49.7, 56.0]**	**74.4 [71.0, 77.8]**	**72.3 [70.4, 74.3]**	58.7 [53.1, 64.2]	59.5 [53.0, 65.9]	61.4 [60.0, 62.8]	20.4 [9.72, 31.0]	8 [−3.6, 19.6]	20.9 [7.47, 34.3]
Lao PDR^a^	**15.0 [12.3, 17.7]**	**90.7 [88.9, 92.5]**	**89.3 [88.2, 90.3]**	**15.9 [13.9, 17.8]**	**78.2 [69.4, 86.9]**	**79.8 [76.0, 83.7]**	**5.98 [****–**.**65, 12.6]**	**26 [12.7, 39.2]**	**32.1 [23.5, 40.6]**
Malawi^a^^b^	**15.3 [13.2, 17.4]**	**37.3 [35.0, 39.7]**	**32.1 [29.8, 34.4]**	41.9 [37.0, 46.7]	34.4 [28.5, 40.2]	13.8 [11.0, 16.6]	25.9 [17.0, 34.7]	10.4 [3.23, 17.5]	10.2 [5.32, 15.2]
Mali^a^^b^	**31.9 [30.0, 33.8]**	**59.8 [56.9, 62.8]**	**85.6 [83.9, 87.4]**	67.1 [61.6, 72.5]	51.2 [44.3, 58.0]	56.9 [51.0, 62.7]	36.8 [27.8, 45.7]	8 [−1.6, 17.7]	19.5 [10.3, 28.6]
Moldova	100 [100, 100]	42.5 [36.0, 49.0]	48.2 [41.8, 54.7]		1.40 [−1.5, 4.4]	2.00 [–.78, 4.78]	0.0 [−16.2, 16.2]	25.6 [11.2, 39.9]	5.3 [−11.6, 22.2]
Mongolia	99.0 [98.5, 99.5]	71.7 [68.8, 74.7]	65.7 [63.3, 68.2]	.819 [−2.0, 3.70]	1.90 [–.19, 4]	1.75 [.135, 3.37]	**1.06 [−4**.**9, 7.04]**	**4.2 [−10.8, 19.2]**	**38.1 [30.5, 45.6]**
Mozambique^a^^b^	**9.67 [7.93, 11.4]**	**68.6 [66.5, 70.7]**	**50.1 [47.7, 52.5]**	63.7 [58.8, 68.5]	52 [46.1, 57.8]	56.4 [51.2, 61.5]	45.3 [36.9, 53.6]	31.3 [23.3, 39.2]	21.5 [12.3, 30.6]
Namibia^b^	99.4 [98.8, 100.]	98.2 [97.3, 99.2]	95.6 [93.4, 97.8]	40.3 [31.0, 49.5]	36.6 [30.4, 42.7]	25.4 [21.8, 29.0]	13.5 [–.18, 27.1]	22.3 [6.07, 38.5]	−23.5 [−30.0, −17.0]
Nepal^a^	56.3 [53.0, 59.7]	89.5 [87.2, 91.8]	21.2 [18.6, 23.7]	**44.6 [38.7, 50.4]**	**55.3 [48.8, 61.7]**	**69.7 [64.8, 74.6]**	27.4 [15.4, 39.3]	25.5 [13.4, 37.5]	−.35 [−8.4, 7.78]
Niger^a^^b^	**8.23 [7.18, 9.29]**	**39.2 [36.9, 41.5]**	**78.0 [76.2, 79.7]**	**44.0 [38.2, 49.8]**	**54.5 [47.0, 61.9]**	**59.3 [53.6, 64.9]**		29.2 [21.0, 37.3]	31.9 [24.3, 39.4]
Nigeria^a^^b^	61.0 [57.1, 65.0]	64.1 [62.6, 65.6]	66.0 [64.6, 67.4]	73.4 [65.9, 80.8]	77.1 [74.1, 80.0]	80.1 [77.1, 83.0]	**37.6 [27.8, 47.3]**	**48.1 [43.5, 52.6]**	**54 [49.9, 58.0]**
Peru^a^	56.1 [54.2, 58.1]	41.9 [39.7, 44.0]	33.2 [31.1, 35.2]	60.4 [58.5, 62.3]	42.8 [37.7, 48.0]	35.1 [29.6, 40.5]	14.3 [−13.0, 41.6]	6.90 [−5.4, 19.2]	5.79 [−6.2, 17.8]
Philippines^a^	47.0 [44.7, 49.2]	49.5 [47.2, 51.8]	39.1 [37.0, 41.3]	65.6 [60.9, 70.2]	68 [63.7, 72.2]	50.6 [45.9, 55.2]	27.5 [18.5, 36.4]	23.5 [15.0, 31.9]	17.5 [8.92, 26.0]
Rwanda^a^^b^	**41.8 [39.2, 44.4]**	**67.7 [65.3, 70.0]**	**47.8 [45.2, 50.5]**	41.5 [35.0, 47.9]	39.2 [33.9, 44.4]	13.0 [11.3, 14.7]	6.40 [−3.2, 16.0]	−.5 [−8.5, 7.56]	8.5 [−71.0, 88.0]
Sao Tome and Principe^a^^b^	71.8 [67.3, 76.2]	71.6 [64.6, 78.6]	79.1 [74.5, 83.8]	17.4 [−2.5, 37.4]	16.1 [5.11, 27.0]	12.5 [7.67, 17.3]	7.56 [−9.1, 24.3]	13.5 [−9.8, 36.8]	4.23 [−4.4, 12.9]
Senegal^a^^b^	97.4 [96.2, 98.5]	81.3 [79.0, 83.7]	85.0 [81.5, 88.5]	63.1 [59.0, 67.3]	69.9 [64.7, 75.0]	55.0 [49.9, 60.2]		6.10 [−4.6, 16.8]	7.19 [−4.9, 19.3]
Sierra Leone^a^^b^	**16.2 [13.0, 19.4]**	**73.2 [70.2, 76.3]**	**67.1 [64.2, 70.0]**	38.9 [29.3, 48.6]	44.4 [37.2, 51.7]	31.7 [24.4, 38.9]	18.4 [7.22, 29.7]	1.59 [−9.4, 12.6]	-11.0 [-22.0, -0.1]
Suriname	69.4 [65.4, 73.3]	48.7 [45.3, 52.1]	46.6 [44.0, 49.1]	21.7 [9.48, 33.9]	14.7 [7.13, 22.2]	12.2 [4.10, 20.2]			
Swaziland^a^^b^	91.7 [89.4, 94.0]	60.7 [56.6, 64.9]		39.8 [31.6, 48.0]	42.2 [34.5, 49.8]	29 [20.0, 37.9]	19.6 [8.84, 30.3]	−3.8 [−16.0, 9.4]	−8.0 [−21.0, 4.9]
Togo^a^^b^	92.4 [90.6, 94.2]	85.6 [83.0, 88.3]	91.0 [89.3, 92.7]	60.1 [50.6, 69.6]	40.5 [33.5, 47.4]	68.5 [65.7, 71.3]	29.8 [26.5, 33.1]	22.9 [11.2, 34.5]	9.59 [1.52, 17.6]
Uganda^a^^b^	**13.1 [11.0, 15.1]**	**56.7 [53.7, 59.8]**	**69.3 [66.2, 72.4]**	**6.39 [−5**.**0, 17.8]**	**47.4 [41.4, 53.3]**	**44.7 [38.7, 50.6]**	−6.8 [−17.0, 3.4]	5.90 [−3.0, 14.8]	3.29 [−6.7, 13.3]
Vietnam^a^	88.3 [87.0, 89.7]	90.4 [88.1, 92.8]	63.8 [60.3, 67.3]		46.6 [33.5, 59.6]	28.3 [23.8, 32.8]	75.0 [60.8, 89.2]	46.3 [35.0, 57.5]	
Zambia^a^^b^	76.5 [73.5, 79.6]	95.0 [93.6, 96.4]	79.3 [77.2, 81.3]	71.3 [66.2, 76.5]	65.3 [59.6, 70.9]	49.1 [46.4, 51.8]	16.1 [3.95, 28.2]	7.59 [−1.9, 17.1]	17.7 [14.0, 21.3]

^a^Countdown country; ^b^Sub-Saharan African country.

Countries with significantly enlarged absolute difference between Q1 and Q5 in round 3 comparing to round 1 are shown in bold.

**Table A9.** Absolute difference between Q5 and Q1 in the nine selected child health interventions by survey round and country, 41 countries (continued).

	Access to improved water (%)			≥4 antenatal care visits (%)			Care seeking for suspected pneumonia (%)		
Country	Round 1	Round 2	Round 3	Round 1	Round 2	Round 3	Round 1	Round 2	Round 3
Armenia	**4.9 [1.6, 8.3]**	**11.5 [6.10, 17.0]**	**23.5 [17.3, 29.7]**	49.6 [38.2, 60.9]	43.5 [30.7, 56.2]	8.39 [2.18, 14.6]	−3.8 [−18.9, 11.3]		
Bangladesh^a^	0.6 [−1.0, 2.2]	−6.1 [−9.0, −3.2]	4.2 [1.0, 7.4]	43.8 [38.7, 48.8]	39.8 [33.8, 45.7]	39.2 [33.4, 45.0]	33.4 [24.9, 41.8]	28.0 [5.59, 50.6]	17.4 [−47.9, 82.8]
Benin^a^^b^	49.8 [46.1, 53.5]	39.5 [37.0, 42.0]	26.0 [23.1, 28.9]	41.7 [34.8, 48.5]	47.8 [43.9, 51.6]	39 [34.3, 43.6]	23 [7.85, 38.1]	20.2 [10.6, 29.7]	11.4 [−5.2, 28.0]
Burkina Faso^a^^b^	42.8 [39.5, 46.0]	15.7 [12.6, 18.7]	32.9 [29.6, 36.1]	19.1 [14.6, 23.5]	23 [18.4, 27.5]	48.6 [34.9, 62.2]	34.3 [14.7, 53.8]		
Burundi^a^^b^	**6.3 [2.0, 10.6]**	**10.2 [6.8, 13.7]**	**16.4 [11.4, 21.5]**			2.90 [−2.6, 8.49]	24.2 [13.2, 35.1]	4.40 [−7.4, 16.2]	6.09 [−5.6, 17.8]
Cambodia^a^	27.1 [23.1, 31.1]		29.7 [24.5, 34.8]	42.1 [36.1, 48.0]	29.1 [24.2, 34.0]	20.6 [11.1, 30.0]	20 [–.42, 40.4]	12.7 [4.46, 20.9]	
Cameroon^a^^b^	**-7.3 [-11.0, -3.6]**	**61.9 [58.9, 64.8]**	**55.4 [51.4, 59.4]**	46.6 [41.2, 52.1]	53.6 [48.2, 58.9]	21.5 [8.03, 34.9]	30.2 [12.2, 48.1]	29.8 [16.3, 43.2]	
Central African Republic^a^^b^	74.2 [72.1, 76.4]	49.2 [46.1, 52.4]	50.2 [47.4, 53.0]		35.7 [28.0, 43.3]	**3.14 [−3**.**2, 9.49]**	**17.8 [3.73, 31.8]**	**36.5 [20.4, 52.5]**	
Colombia	**40.7 [37.7, 43.7]**	**44.3 [42.4, 46.3]**	**45.6 [43.8, 47.4]**	32.3 [26.3, 38.2]	27.8 [24.0, 31.5]	18.7 [15.7, 21.6]	31.9 [14.8, 48.9]	−18.2 [−30.1, −6.3]	13 [−5.4, 31.4]
Congo, Dem. Rep.^a^^b^	92.1 [91.0, 93.2]	71.8 [66.7, 77.0]	74.4 [71.3, 77.5]	20.3 [12.6, 28.1]	29.0 [23.7, 34.4]	8.54 [3.20, 13.8]	14.6 [2.79, 26.4]	6.3 [−12.5, 25.1]	
Cote d’Ivoire^a^^b^	**20.0 [19.2, 20.9]**	**47.4 [44.9, 49.9]**	**36.4 [30.9, 42.0]**			45.1 [38.1, 52.0]	15.0 [10.3, 19.7]	50.9 [28.2, 73.5]	23.9 [4.45, 43.3]
Dominican Republic	13.3 [10.7, 15.8]	8.65 [5.33, 11.9]	11.6 [7.66, 15.6]	9.5 [5.83, 13.1]	8 [4.23, 11.7]	6.81 [3.20, 10.4]	−16.1 [−28.5, −3.7]	1.9 [−20.8, 24.7]	3.0 [−15.7, 21.7]
Egypt^a^	21.8 [20.0, 23.6]	4.87 [3.54, 6.20]	5.39 [3.75, 7.03]	55.5 [51.1, 59.8]	46.6 [42.8, 50.3]	21.6 [19.0, 24.1]	21.5 [9.81, 33.1]	11.9 [.798, 23.0]	1.21 [−7.0, 9.47]
Ethiopia^a^^b^	61.8 [58.5, 65.1]	30.1 [26.3, 33.9]	62.5 [58.8, 66.2]	29 [22.6, 35.3]	33.6 [27.9, 39.2]	37.8 [31.4, 44.1]	22.3 [12.7, 31.8]	14.5 [2.73, 26.2]	46.2 [27.5, 64.8]
Ghana^a^^b^	36.9 [32.9, 40.8]	34.8 [30.4, 39.3]	28.0 [23.6, 32.5]	38.3 [31.0, 45.5]	32.2 [24.8, 39.5]	21.6 [−15.7, 58.9]	37.8 [17.0, 58.5]	8.1 [−18.8, 35.0]	
Haiti^a^	61.0 [54.9, 67.2]	57.4 [53.0, 61.7]	68.7 [64.7, 72.7]	47.2 [40.1, 54.2]	48.4 [41.2, 55.5]	36.7 [29.9, 43.4]	15.7 [3.31, 28.0]	16.6 [.350, 32.8]	26.5 [15.7, 37.2]
Indonesia^a^	56.9 [54.4, 59.4]	28.1 [25.8, 30.4]	25.1 [23.1, 27.1]	32.7 [26.3, 39.0]	36.5 [32.6, 40.3]	27.6 [24.3, 30.8]	25.5 [9.28, 41.7]	23.4 [12.1, 34.6]	10.3 [−6.7, 27.3]
Jordan	4.26 [3.09, 5.44]	5.57 [4.40, 6.75]	3.23 [2.25, 4.21]	10.8 [6.17, 15.4]	7.69 [4.37, 11.0]	10.8 [3.91, 17.6]		−6.4 [−30.5, 17.7]	
Kenya^a^^b^	59.4 [56.0, 62.8]	58.4 [54.6, 62.2]	48.2 [46.0, 50.4]	25.7 [18.7, 32.6]	26.5 [20.1, 32.8]	30.9 [10.6, 51.3]	20.1 [9.48, 30.7]	6.1 [−24.2, 36.4]	10.2 [2.25, 18.1]
Lao PDR^a^	**26.5 [22.8, 30.1]**	**51.1 [47.5, 54.7]**	**40.7 [38.7, 42.6]**			73.5 [68.5, 78.4]	0.7 [−19.8, 21.2]	43.4 [21.1, 65.6]	
Malawi^a^^b^	37.2 [34.0, 40.5]	30.1 [27.8, 32.4]	17.3 [15.4, 19.3]	17.7 [12.6, 22.7]		15.8 [4.56, 27.0]	14.1 [2.44, 25.7]	4.84 [.131, 9.55]	
Mali^a^^b^	55.5 [53.5, 57.5]	56.5 [54.2, 58.9]	59.6 [57.1, 62.1]	48.5 [42.8, 54.1]	40.6 [33.5, 47.6]	46.1 [40.9, 51.2]	42.8 [32.1, 53.4]	32.6 [20.7, 44.4]	22 [1.24, 42.7]
Moldova	**13.3 [9.58, 17.0]**	**7.61 [2.16, 13.0]**	**32.9 [26.9, 38.8]**		.800 [−7.7, 9.34]	−5.7 [−52.8, 41.4]	8.2 [−12.5, 28.9]		
Mongolia	77.7 [75.6, 79.8]	64.7 [61.5, 67.8]	56.9 [54.3, 59.5]			7.3 [−11.1, 25.7]	16.9 [–.20, 34.0]	14.2 [5.24, 23.1]	
Mozambique^a^^b^	78.2 [75.9, 80.5]	47.1 [44.4, 49.8]	61.2 [58.4, 64.0]	37.3 [30.9, 43.6]	28.1 [22.4, 33.7]	20.3 [8.07, 32.5]	12.9 [−8.4, 34.2]	22.6 [−3.9, 49.3]	
Namibia^b^	49.8 [45.4, 54.2]	32.0 [28.7, 35.3]	38.1 [34.8, 41.4]	19.9 [10.7, 29.0]	14.8 [7.65, 21.9]	12.6 [−38.4, 63.6]	26 [4.23, 47.7]	30.5 [5.85, 55.2]	
Nepal^a^	37.1 [34.1, 40.1]	39.4 [36.3, 42.6]	5.56 [2.83, 8.28]	41.4 [35.6, 47.1]	48.6 [40.4, 56.7]	14.1 [5.04, 23.1]	18 [−2.3, 38.3]	29.9 [20.9, 38.8]	
Niger^a^^b^	62.3 [59.7, 64.9]	81.8 [79.4, 84.2]	55.1 [52.5, 57.6]	25.3 [21.0, 29.5]	21.6 [16.7, 26.4]	26.4 [13.9, 38.8]	25.3 [9.71, 40.8]		
Nigeria^a^^b^	67.3 [63.8, 70.7]	58.1 [56.4, 59.8]	42.8 [40.9, 44.7]	64.8 [55.7, 73.8]	65 [61.2, 68.7]	66.6 [63.0, 70.1]	42.1 [25.4, 58.7]	34.3 [18.2, 50.5]	36.9 [16.9, 56.8]
Peru^a^	42.9 [40.2, 45.5]	30.6 [27.9, 33.3]	21.1 [18.4, 23.8]	18.8 [−8.9, 46.6]	15.6 [11.1, 20.0]	11.3 [8.21, 14.3]	6.49 [−1.5, 14.5]	2.3 [−16.6, 21.2]	4.1 [−25.7, 33.9]
Philippines^a^	13.8 [12.0, 15.6]	14.7 [13.0, 16.4]	10.8 [9.54, 12.1]	36.4 [30.9, 41.8]	32 [27.0, 36.9]	23.2 [18.8, 27.5]	25 [9.57, 40.4]	22.8 [.553, 45.0]	26.6 [4.77, 48.4]
Rwanda^a^^b^	66.4 [63.8, 68.9]	57.0 [54.3, 59.7]	29.3 [26.4, 32.1]	10 [6.01, 13.9]	10.4 [6.73, 14.0]	3.2 [−15.5, 21.9]	20.2 [12.2, 28.1]	20.3 [12.2, 28.3]	26.5 [4.80, 48.3]
Sao Tome and Principe^a^^b^	12.6 [7.78, 17.5]	7.64 [5.33, 9.95]	7.23 [4.93, 9.52]	32.3 [21.4, 43.1]	−3.5 [−20.4, 13.4]	12.1 [4.77, 19.5]			
Senegal^a^^b^	77.2 [75.1, 79.3]	58.3 [55.9, 60.7]	37.8 [35.0, 40.6]	33.9 [28.0, 39.7]	42.5 [12.3, 72.6]	9.54 [.486, 18.6]	26.6 [13.2, 39.9]	11.6 [−21.6, 44.8]	
Sierra Leone^a^^b^	85.8 [82.9, 88.6]	79.7 [77.1, 82.3]	59.2 [56.3, 62.0]	18.2 [9.43, 26.9]	4.89 [−2.3, 12.1]	3.94 [−6.0, 13.8]	6.5 [−18.5, 31.5]	7.4 [−15.2, 30.0]	
Suriname	63.8 [59.5, 68.1]	31.8 [28.3, 35.3]	24.3 [22.1, 26.5]		10.9 [.074, 21.7]	4.4 [−26.8, 35.6]			
Swaziland^a^^b^	82.9 [80.2, 85.5]	62.9 [58.4, 67.4]		13.2 [5.83, 20.5]	13 [4.42, 21.5]	9.13 [−4.4, 22.7]	4.1 [−20.2, 28.4]	1.4 [−17.5, 20.3]	
Togo^a^^b^	88.3 [85.9, 90.6]	69.2 [65.6, 72.8]	42.7 [39.4, 46.1]		34.6 [9.21, 60.1]	4.25 [−8.3, 16.8]	9.30 [−3.6, 22.2]	4.8 [−11.7, 21.3]	
Uganda^a^^b^	35.1 [31.3, 39.0]	17.9 [14.6, 21.1]	12.5 [9.29, 15.8]	**1.79 [−5**.**4, 9.08]**	**20.3 [14.4, 26.1]**	**16.2 [10.2, 22.1]**	13.3 [1.17, 25.4]	−2.0 [−16.1, 12.1]	4.5 [−8.2, 17.2]
Vietnam^a^	53.1 [51.5, 54.8]	27.5 [23.2, 31.7]	24.2 [21.2, 27.3]			−23.5 [−37.0, −10.0]	6.55 [2.63, 10.4]		
Zambia^a^^b^	92.7 [90.8, 94.5]	84.4 [82.0, 86.8]	62.8 [60.6, 65.1]	21.2 [15.1, 27.2]	.899 [−6.1, 7.89]	14.3 [−10.6, 39.2]	10.2 [−2.9, 23.3]	−21.7 [−44.0, 0.6]	5.92 [−4.7, 16.5]

^a^Countdown country; ^b^Sub-Saharan African country.

Countries with significantly enlarged absolute difference between Q1 and Q5 in round 3 compared with round 1 are shown in bold.

**Table A9.** Absolute difference between Q5 and Q1 in the nine selected child health interventions by survey round and country, 41 countries (continued).

	Received ORT for diarrhea (%)			Received vitamin A supplement (%)			ITN for children (%)		
Country	Round 1	Round 2	Round 3	Round 1	Round 2	Round 3	Round 1	Round 2	Round 3
Armenia		20.3 [−1.4, 42.0]							
Bangladesh^a^	20.9 [3.16, 38.6]	13.7 [−1.8, 29.2]	11.6 [−8.5, 31.7]	8.70 [4.05, 13.3]	−1 [−4.8, 2.82]	12.8 [9.72, 15.8]			
Benin^a^^b^	7.5 [−4.9, 19.9]	16.5 [8.13, 24.8]	14.5 [2.03, 26.9]	28.4 [22.8, 33.9]	25 [19.7, 30.2]	23.5 [18.5, 28.4]		24.6 [21.6, 27.5]	3.90 [–.06, 7.86]
Burkina Faso^a^^b^	18.4 [11.1, 25.6]	17.8 [8.43, 27.1]	18.6 [11.3, 25.8]	23.1 [17.8, 28.3]	21.2 [7.37, 35.0]	11.4 [5.80, 16.9]	5.4 [3.46, 7.33]	21.7 [17.1, 26.2]	6.59 [1.36, 11.8]
Burundi^a^^b^	.259 [−7.7, 8.22]	13.3 [4.51, 22.0]	6.40 [−2.2, 15.0]	.204 [−3.1, 3.51]		4.19 [–.25, 8.65]	**10.6 [8.58, 12.7]**	**14.6 [11.0, 18.1]**	**24.2 [18.1, 30.2]**
Cambodia^a^	30.1 [21.1, 39.0]	−1.5 [−9.1, 6.15]	−13.1 [−30.9, 4.7]	27.2 [20.9, 33.4]	−.20 [−6.2, 5.79]	−1.4 [−6.6, 3.84]		−6.6 [−8.8, −4.3]	
Cameroon^a^^b^	26.8 [16.3, 37.2]	29.3 [19.1, 39.4]	28.5 [21.0, 35.9]	19.4 [13.5, 25.2]	−15.5 [−22.1, −8.9]	−9.4 [−15.8, −3.0]	**2 [1.17, 2.82]**	**8.8 [4.71, 12.8]**	**7.90 [5.17, 10.6]**
Central African Republic^a^^b^	13.6 [9.25, 18.0]	22 [13.9, 30.0]	16.6 [9.12, 24.0]	**2.66 [****–**.**23, 5.57]**	**−1.7 [−9**.**3, 5.94]**	**17.8 [12.5, 23.0]**	34.1 [31.6, 36.7]	23.3 [16.8, 29.7]	−1.2 [−7.6, 5.04]
Colombia	−7.9 [−17.7, 1.9]	4.30 [−8.0, 16.6]	14 [–.07, 28.0]						
Congo, Dem. Rep.^a^^b^	16.0 [10.9, 21.0]	12.6 [1.74, 23.4]	11.1 [2.81, 19.3]	**6.75 [5.02, 8.47]**	**28.6 [20.0, 37.1]**	**27.6 [22.6, 32.5]**	7.99 [6.09, 9.88]	8.80 [6.33, 11.2]	4.09 [−2.5, 10.7]
Cote d’Ivoire^a^^b^	29.8 [25.7, 33.8]	17 [8.61, 25.3]	17.5 [9.30, 25.6]	**−1.7 [−2**.**9**, **–**.**55]**	**−3.2 [−12.6, 6.2]**	**26.4 [18.6, 34.1]**	2.86 [1.87, 3.86]	5.90 [3.74, 8.05]	−16.8 [−24.0, −9.5]
Dominican Republic	**-22.1 [-30.6, -13.6]**	**−3.6 [−15.9, 8.7]**	**10.3 [−6**.**6, 27.2]**	11.5 [6.57, 16.4]	6.20 [1.23, 11.1]	5.60 [−3.3, 14.5]			
Egypt^a^	−22.0 [−32.2, −11.8]	−11.0 [−20.5, −1.5]	−4.6 [−14.7, 5.5]	6.8 [3.73, 9.86]	4.7 [2.31, 7.08]	5.1 [2.67, 7.52]			
Ethiopia^a^^b^	25 [15.7, 34.2]	24.3 [15.6, 32.9]	27.3 [15.9, 38.6]	15.8 [8.45, 23.3]	16.3 [9.65, 22.9]	12.9 [5.74, 20.0]		1.5 [.196, 2.80]	
Ghana^a^^b^	24.8 [8.87, 40.7]	4.3 [−13.5, 22.1]	3.3 [−35.2, 41.8]	8.79 [2.48, 15.1]	11 [1.43, 20.5]	7.50 [−4.6, 19.6]	−1.3 [−4.4, 1.88]	−1.9 [−10.3, 6.5]	
Haiti^a^	15.3 [5.54, 25.0]	21 [4.35, 37.6]	10.6 [–.84, 22.0]	1.8 [−10.0, 13.6]	5.00 [−2.3, 12.3]	4.80 [−1.6, 11.2]			20.9 [16.9, 24.8]
Indonesia^a^	−11.5 [−23.2, 0.2]	−4.2 [−12.6, 4.2]	−5.0 [−13.6, 3.6]	17.1 [11.8, 22.3]	19.7 [14.9, 24.4]	12.1 [8.06, 16.1]		−4.9 [−6.3, −3.4]	
Jordan	−20.0 [−27.5, −12.5]	11.6 [.083, 23.1]	−0.8 [−12.7, 11.1]		.099 [−2.5, 2.76]	3.39 [−1.0, 7.87]			
Kenya^a^^b^	6.00 [−4.2, 16.2]	−5.9 [−22.2, 10.4]	5.0 [−39.7, 49.7]	7.30 [.861, 13.7]	6.29 [–.15, 12.7]	12.1 [9.64, 14.5]	**12.4 [9.41, 15.3]**	**16.5 [7.72, 25.2]**	**22.1 [20.2, 23.9]**
Lao PDR^a^	21.3 [5.39, 37.2]	41.3 [19.7, 62.8]	34.3 [18.8, 49.7]	**1.89 [−1**.**6, 5.42]**	**5.30 [****–**.**49, 11.0]**	**17 [10.6, 23.3]**	18.1 [15.4, 20.9]	-9.1 [-17.7, -0.5]	−23.0 [−28.0, −18.0]
Malawi^a^^b^	10.3 [1.71, 19.0]	4.19 [−2.9, 11.3]	8.39 [1.62, 15.1]	7.40 [2.52, 12.2]	2.10 [−1.6, 5.84]		27.8 [22.9, 32.6]	27.1 [23.2, 30.9]	
Mali^a^^b^	21.9 [15.3, 28.4]	21.1 [12.9, 29.2]	22.3 [12.0, 32.5]	22.1 [14.8, 29.3]	10.5 [3.71, 17.2]	17.7 [11.2, 24.1]		6.59 [.991, 12.2]	1.99 [−2.8, 6.89]
Moldova	5.06 [−5.4, 15.6]		−12.4 [−48.0, 23.2]			−1.5 [−8.6, 5.54]			
Mongolia	19.7 [7.26, 32.1]	17.9 [−1.7, 37.5]	3.1 [−10.0, 16.3]	5.92 [2.42, 9.42]	15.2 [8.21, 22.1]	4.89 [.771, 9.01]			
Mozambique^a^^b^	25.2 [14.1, 36.2]	10.5 [1.23, 19.7]	28.8 [16.9, 40.6]	31.3 [25.7, 36.8]	19.3 [14.2, 24.3]	24.5 [19.8, 29.1]			5.29 [–.08, 10.6]
Namibia^b^	21.2 [−1.3, 43.7]	8.59 [−7.5, 24.7]	5.5 [−34.5, 45.5]	−.70 [−8.6, 7.28]	−11.8 [−18.2, −5.3]	−13.5 [−24.0, −3.0]		−10.2 [−13.3, −7.0]	−2.2 [−15.0, 10.6]
Nepal^a^	21.1 [10.5, 31.6]	21.7 [7.96, 35.4]	−3.7 [−18.6, 11.2]		−.09 [−5.9, 5.70]				
Niger^a^^b^		17.9 [11.6, 24.1]	15.2 [5.85, 24.5]		13.7 [6.91, 20.6]	21.0 [15.4, 26.7]		7.9 [4.72, 11.0]	21.8 [17.6, 25.9]
Nigeria^a^^b^	25.1 [11.5, 38.6]	37.7 [27.5, 47.8]	32.6 [24.5, 40.6]	42.8 [33.5, 52.0]	31.3 [27.9, 34.6]	46.3 [41.5, 51.2]	**−1.1 [−2**.**1**, **–**.**02]**	**5.40 [3.92, 6.87]**	**6.00 [3.16, 8.83]**
Peru^a^	28.2 [18.9, 37.4]	40.8 [25.8, 55.7]	9.9 [−3.0, 22.8]	1.6 [−9.5, 12.7]	−5.4 [−6.9, −3.8]	−8.5 [−10.3, −6.7]			
Philippines^a^	20 [6.67, 33.3]	17.4 [.673, 34.1]	5.6 [−10.3, 21.5]	22.9 [18.2, 27.5]	7.59 [1.83, 13.3]	−.19 [−4.7, 4.32]			
Rwanda^a^^b^	9.90 [3.18, 16.6]	7.49 [.838, 14.1]	15 [1.73, 28.2]	3.79 [−4.1, 11.7]	3.09 [−1.0, 7.22]	−2.7 [−5.0, –.32]		25.4 [21.7, 29.1]	29.5 [28.2, 30.7]
Sao Tome and Principe^a^^b^	4.8 [−11.4, 21.0]	−3.2 [−26.0, 19.6]	.690 [−1.2, 2.65]	16.4 [5.77, 27.0]		49.0 [43.0, 55.0]	22.1 [7.67, 36.5]		
Senegal^a^^b^		12.7 [6.03, 19.3]	−0.4 [−13.8, 12.9]	22.9 [−17.1, 62.9]	5.40 [–.40, 11.2]		−4.1 [−6.7, −1.5]	1.7 [–.32, 3.72]	−2.2 [−15., 10.6]
Sierra Leone^a^^b^	41.5 [31.8, 51.1]	26.6 [10.2, 42.9]	1.20 [−6.8, 9.26]	**−10.6 [−16.0, −5.2]**	**8.5 [3.19, 13.8]**	.**700****[−5**.**0, 6.49]**	−1.8 [−5.6, 2.01]	4.9 [−1.2, 11.0]	−16.0 [−22.5, −9.5]
Suriname	35.5 [5.82, 65.2]								
Swaziland^a^^b^	−2.7 [−10.1, 4.7]	−5.4 [−21.1, 10.3]	2.3 [−14.9, 19.5]	3.31 [1.14, 5.47]	−5.0 [−12.2, 2.2]	6.09 [−1.9, 14.1]	.150 [–.16, .469]		−1.3 [−2.8, .200]
Togo^a^^b^	−4.5 [−6.6, −2.5]	10.3 [.282, 20.3]	−3.5 [−16.3, 9.3]	−2.0 [−7.9, 3.80]	7.19 [.199, 14.2]	−3.5 [−6.6, –.32]	8.20 [6.23, 10.1]	−5.0 [−12.2, 2.2]	0.1 [−30.8, 31.0]
Uganda^a^^b^	5.6 [−3.8, 15.0]	−8.5 [−17.5, .0.5]	2.50 [−7.9, 12.9]	−9.4 [−16.8, −2.0]	−2.2 [−7.8, 3.48]	−5.6 [−11.7, 0.5]		3.5 [–.18, 7.18]	−.79 [−6.6, 5.07]
Vietnam^a^	22.8 [1.67, 44.0]		11.7 [−8.4, 31.8]	16.8 [9.13, 24.5]	13.7 [4.01, 23.3]		−2.1 [−3.9, –.41]		
Zambia^a^^b^	10.2 [−1.6, 22.0]	−0.6 [−18.1, 16.9]	8.40 [−1.1, 17.9]	21.6 [15.4, 27.7]	1.5 [−7.2, 10.2]	12.1 [6.51, 17.6]	11.4 [7.92, 14.8]	11.6 [5.23, 17.9]	.200 [−6.8, 7.23]

^a^Countdown country; ^b^Sub-Saharan African country.

Countries with significantly enlarged absolute difference between Q1 and Q5 in round 3 comparing to round 1 are shown in bold.

**Table A10.** Concentration index of three child health outcomes by survey round and country, 41 countries.

	Infant mortality per 1,000 live births			Under-five mortality per 1,000 live births			Stunting prevalence (%)		
Country	Round 1	Round 2	Round 3	Round 1	Round 2	Round 3	Round 1	Round 2	Round 3
Armenia	−9.8 [−20.0, 0.33]	−12.6 [−29.0, 3.8]	−5.9 [−16.3, 4.3]	−11.9 [−20.7, −3.0]	−11.7 [−26.0, 2.5]	−10.1 [−19.2, −1.1]	−12.5 [−22.9, −2.1]	5.1 [−6.8, 17.0]	−7.2 [−17.5, 3.1]
Bangladesh^a^	−6.9 [−12.1, −1.8]	−12.0 [−19.7, −4.3]	−12.0 [−21.0, −3.0]	−10.3 [−15.0, −5.6]	−12.2 [−21.9, −2.5]	−12.2 [−19.8, −4.6]	−11.3 [−14.5, −8.1]	−12.9 [−16.8, −9.1]	−16.1 [−19.8, −12.5]
Benin^a^^b^	−11.6 [−23.1, −0.20]	−5.1 [−13.4, 3.2]	−7.8 [−15.5, −0.17]	−11.5 [−21.3, −1.8]	−7.7 [−17.5, 1.9]	−10.5 [−19.9, −1.0]	−9.5 [−14.2, −4.8]	−9.3 [−11.8, −6.8]	−5.4 [−8.6, −2.3]
Burkina Faso^a^^b^	−4.6 [−8.3, −0.87]		−9.6 [−15.6, −3.6]	−5.3 [−11.2, 0.44]		−10.2 [−16.8, −3.6]	−8.9 [−11.8, −5.9]	−12.9 [−16.8, −9.0]	−10.8 [−14.7, −7.0]
Burundi^a^^b^			−11.1 [−18.6, −3.5]			−10.7 [−18.9, −2.6]	−7.6 [−9.6, −5.6]		−8.2 [−12.2, −4.3]
Cambodia^a^	−9.7 [−20.3, 0.81]	−11.4 [−28.1, 5.2]	−25.6 [−36.2, −15.0]	−11.9 [−22.3, −1.4]	−12.6 [−28.1, 2.9]	−26.0 [−37.2, −14.7]	−8.3 [−12.4, −4.1]	−12.4 [−16.6, −8.1]	−14.4 [−19.3, −9.6]
Cameroon^a^^b^	−11.7 [−19.0, −4.5]		−10.2 [−15.3, −5.1]	−12.8 [−21.1, −4.5]		−17.1 [−24.8, −9.4]	−15.6 [−20.6, −10.6]	−22.0 [−25.5, −18.4]	−22.6 [−27.1, −18.1]
Central African Republic^a^^b^							**−4.4 [−5**.**5**, **−3**.**2]**	**−5.5 [−8**.**8**, **−2**.**2]**	**−9.2 [−12.2**, **−6**.**2]**
Colombia	−15.9 [−24.7, −7.1]	−15.2 [−22.7, −7.7]	−7.1 [−13.0, −1.2]	−17.0 [−24.3, −9.8]	−17.1 [−25.4, −8.8]	−11.5 [−18.3, −4.7]	−21.9 [−28.0, −15.8]	−23.9 [−27.4, −20.4]	−18.3 [−21.6, −14.9]
Congo, Dem. Rep.^a^^b^		−9.6 [−18.7, −0.60]	−0.88 [−7.8, 6.1]		−9.5 [−18.4, −0.59]	−3.9 [−12.5, 4.6]	−9.1 [−10.4, −7.8]	−6.3 [−11.0, −1.6]	−11.6 [−14.7, −8.6]
Cote d’Ivoire^a^^b^			−5.2 [−12.1, 1.5]			−5.7 [−12.9, 1.4]		−12.5 [−15.5, −9.6]	−15.2 [−21.1, −9.4]
Dominican Republic	−14.9 [−24.0, −5.9]	−11.6 [−17.0, −6.2]	−13.2 [−29.7, 3.1]	−16.6 [−26.2, −7.0]	−14.4 [−20.3, −8.6]	−13.4 [−27.8, 1.0]	−24.5 [−28.8, −20.3]	−22.9 [−27.8, −18.1]	−25.3 [−34.6, −16.0]
Egypt^a^	−17.0 [−25.5, −8.4]	−15.7 [−24.2, −7.1]	−12.9 [−19.1, −6.8]	−18.6 [−28.7, −8.5]	−16.3 [−24.9, −7.7]	−14.4 [−21.5, −7.3]	−14.5 [−18.1, −10.9]	−2.1 [−5.6, 1.3]	−1.8 [−5.1, 1.5]
Ethiopia^a^^b^	−0.33 [−10.0, 9.3]	−3.7 [−11.4, 3.9]	−9.4 [−11.1, −7.7]	−1.2 [−11.6, 9.0]	−4.5 [−14.0, 4.9]	−9.9 [−13.1, −6.6]	−3.2 [−5.7, −0.81]	−4.0 [−8.0, 0.00]	−6.5 [−9.3, −3.6]
Ghana^a^^b^	−0.75 [−3.8, 5.3]	−3.7 [−10.5, 2.9]	−3.3 [−9.4, 2.6]	−5.7 [−10.3, −1.1]	−8.4 [−16.3, −0.51]	−10.0 [−13.2, −6.7]	−15.3 [−20.4, −10.1]	−13.7 [−20.7, −6.8]	−21.6 [−28.9, −14.3]
Haiti^a^	−0.25 [−6.7, 6.2]	−7.6 [−16.5, 1.2]	−0.51 [−5.9, 6.9]	−7.1 [−10.9, −3.3]	−10.9 [−21.3, −0.57]	−4.5 [−12.8, 3.7]	−18.1 [−22.2, −14.0]	−22.0 [−28.0, −15.9]	−21.1 [−26.9, −15.4]
Indonesia^a^	−17.8 [−32.6, −2.9]	−16.2 [−21.1, −11.3]	−18.4 [−28.5, −8.2]	−18.0 [−32.6, −3.3]	−18.7 [−25.0, −12.3]	−20.1 [−29.3, −11.0]			
Jordan	−7.2 [−12.1, −2.3]	−8.1 [−18.6, 2.2]	−11.0 [−25.4, 3.4]	−7.5 [−13.2, −1.7]	−10.6 [−21.1, −0.21]	−9.2 [−19.8, 1.2]	−17.0 [−23.2, −10.8]	−9.3 [−15.6, −2.9]	−25.9 [−32.2, −19.6]
Kenya^a^^b^	−10.0 [−15.3, −4.8]	−5.1 [−10.4, 0.06]	−0.49 [−2.9, 3.8]	−11.2 [−16.8, −5.6]	−9.6 [−15.5, −3.6]	−2.3 [−6.9, 2.2]	**−10.4 [−14.7**, **−6**.**0]**	**−11.7 [−16.1**, **−7**.**3]**	**−17.3 [−19.8**, **−1****4.8]**
Lao PDR^a^							**−5.4 [−11.0, 0.09]**	**−19.2 [−23.3**, **−1****5.1]**	**−23.7 [−26.2**, **−2****1.1]**
Malawi^a^^b^	−7.1 [−13.5, −0.79]			−7.4 [−13.9, −0.96]			−6.9 [−9.4, −4.3]	−5.5 [−7.2, −3.7]	−8.5 [−10.7, −6.3]
Mali^a^^b^	−4.7 [−12.6, 3.0]	−6.1 [−13.1, 0.82]	−6.5 [−14.6, 1.5]	−5.5 [−16.0, 4.8]	−7.9 [−19.7, 3.7]	−10.3 [−21.8, 1.1]	−11.0 [−13.8, −8.3]	−9.1 [−11.9, −6.2]	−12.8 [−17.3, −8.3]
Moldova		−5.3 [−13.5, 2.8]			−10.0 [−18.3, −1.7]			−14.7 [−26.9, −2.4]	−25.8 [−41.3, −10.3]
Mongolia							**−15.3 [−19.4**, **−1****1.1]**	**−13.8 [−19.5**, **−8**.**0]**	**−27.1 [−33.3**, **−2****0.8]**
Mozambique^a^^b^	−12.5 [−21.3, −3.7]		−5.7 [−8.7, −2.7]	−10.6 [−19.9, −1.3]		−6.6 [−10.6, −2.7]	−10.1 [−12.9, −7.3]	−16.5 [−19.2, −13.8]	−10.3 [−13.3, −7.2]
Namibia^b^	−10.8 [−22.3, 0.54]	−8.2 [−22.1, 5.5]	−11.3 [−21.3, −1.3]	−9.8 [−25.6, 5.9]	−10.9 [−25.4, 3.5]	−9.0 [−21.0, 2.9]	−13.9 [−19.3, −8.5]	−14.2 [−19.6, −8.7]	−12.7 [−21.7, −3.6]
Nepal^a^	−7.6 [−14.4, −0.91]	−9.2 [−17.3, −1.1]		−11.1 [−18.3, −3.9]	−11.5 [−20.9, −2.0]		**−8.3 [−11.2**, **−5**.**5]**	**−11.2 [−14.7**, **−7**.**7]**	**−22.5 [−26.4**, **−1****8.7]**
Niger^a^^b^		−6.2 [−15.3, 2.8]	−3.9 [−9.7, 1.7]		−5.3 [−14.9, 4.2]	−4.8 [−13.6, 3.8]	−6.4 [−10.5, −2.4]	−4.8 [−8.8, −0.89]	−4.0 [−8.0, 0.02]
Nigeria^a^^b^	−15.2 [−27.3, −3.1]	−9.0 [−16.2, −1.9]	−11.7 [−18.7, −4.6]	−16.7 [−33.0, −0.40]	−14.3 [−24.9, −3.8]	−17.5 [−26.9, −8.1]	−15.7 [−19.8, −11.5]	−12.7 [−14.7, −10.7]	−18.0 [−19.9, −16.0]
Peru^a^	−23.0 [−40.5, −5.6]	−20.3 [−36.9, −3.8]	−15.0 [−22.2, −7.8]	−23.4 [−40.9, −5.9]	−20.0 [−35.1, −4.8]	−17.0 [−25.3, −8.6]	−37.6 [−43.9, −31.3]	−35.7 [−39.2, −32.1]	−41.9 [−45.7, −38.0]
Philippines^a^	−14.9 [−20.1, −9.8]	−16.1 [−24.3, −8.0]	−18.4 [−26.1, −10.6]	−22.0 [−30.0, −14.1]	−20.1 [−29.6, −10.7]	−22.4 [−29.4, −15.4]			
Rwanda^a^^b^	−8.7 [−14.2, −3.1]	−5.4 [−14.5, 3.6]	−10.5 [−20.6, −0.39]	−9.1 [−14.3, −3.9]	−7.4 [−16.8, 2.0]	−12.8 [−21.4, −4.3]	−7.7 [−10.9, −4.5]	−8.1 [−12.1, −4.1]	−15.5 [−20.5, −10.5]
Sao Tome and Principe^a^^b^		−4.4 [−17.4, 8.5]			−8.0 [−24.7, 8.5]		−12.6 [−20.2, −5.1]	−12.9 [−21.9, −3.8]	−22.6 [−32.1, −13.1]
Senegal^a^^b^		−12.2 [−22.0, −2.5]	−10.7 [−19.3, −2.2]		−15.6 [−27.3, −3.9]	−14.6 [−26.4, −2.8]	−14.6 [−18.0, −11.2]	−23.9 [−30.5, −17.3]	−20.9 [−25.7, −16.1]
Sierra Leone^a^^b^		−8.5 [−15.4, −1.7]	−2.5 [−4.3, −0.61]		−6.3 [−12.4, −0.15]	−3.7 [−8.0, 0.57]	−8.5 [−11.3, −5.6]	−5.9 [−12.8, 0.88]	−6.1 [−10.8, −1.4]
Suriname							**34.7 [27.9, 41.5]**	**−27.6 [−38.1**, **−1****7.2]**	**−26.2 [−35.4**, **−1****7.0]**
Swaziland^a^^b^		−0.66 [−5.2, 6.6]			−1.4 [−6.1, 3.2]		−12.5 [−17.7, −7.2]	−12.3 [−19.9, −4.6]	−22.3 [−28.6, −15.9]
Togo^a^^b^			−7.7 [−18.0, 2.5]			−13.9 [−25.9, −1.9]		−10.6 [−16.0, −5.1]	−15.3 [−21.2, −9.4]
Uganda^a^^b^	**−0.60 [−2**.**4, 3.6]**	**−7.9 [−12.9**, **−2**.**8]**	**−8.2 [−13.6**, **−2**.**8]**	−0.97 [−3.5, 5.5]	−6.3 [−11.2, −1.3]	−10.1 [−16.9, −3.2]	−6.8 [−10.4, −3.2]	−6.8 [−12.9, −0.78]	−6.6 [−13.6, 0.24]
Vietnam^a^							−11.8 [−14.1, −9.6]		
Zambia^a^^b^	−8.5 [−19.0, 2.0]	−0.40 [−6.8, 7.6]	−5.1 [−9.1, −1.0]	−7.1 [−19.0, 4.8]	−0.52 [−8.3, 7.3]	−8.7 [−14.1, −3.2]	−9.0 [−12.1, −5.9]	−4.9 [−8.7, −1.1]	−7.9 [−10.6, −5.2]

^a^Countdown country; ^b^Sub-Saharan African country.

Countries whose concentration index became significantly father away from zero between round 1 and round 3 are shown in bold.

**Table A11.** Concentration index of three selected child health interventions by survey round and country, 41 countries.

	Access to improved sanitation (%)			Delivered with SBA (%)			Received full immunization (%)		
Country	Round 1	Round 2	Round 3	Round 1	Round 2	Round 3	Round 1	Round 2	Round 3
Armenia	**−0.16 [−0**.**61, 0.28]**	**5.3 [4.3, 6.3]**	**19.5 [17.6, 21.5]**	1.4 [0.81, 2.0]	0.43 [0.12, 0.73]	−0.07 [−0.23, 0.07]	2.4 [−2.0, 6.9]	1.3 [−4.5, 7.1]	−2.4 [−5.4, 0.59]
Bangladesh^a^	24.2 [23.1, 25.3]	30.5 [28.6, 32.3]	18.2 [17.2, 19.3]	47.5 [32.6, 62.4]	43.5 [33.5, 53.5]	25.4 [13.3, 37.5]	7.8 [5.9, 9.7]	3.1 [1.5, 4.6]	5.0 [3.6, 6.4]
Benin^a^^b^	64.0 [62.4, 65.6]	56.2 [55.1, 57.3]	61.6 [60.4, 62.8]	13.0 [8.3, 17.8]	9.9 [5.8, 14.1]	8.0 [4.3, 11.7]	8.5 [5.4, 11.6]	11.6 [9.3, 13.9]	2.1 [−0.96, 5.2]
Burkina Faso^a^^b^	65.7 [64.7, 66.8]	79.4 [76.5, 82.3]	66.9 [65.7, 68.2]	15.5 [6.7, 24.3]	0.91 [−6.4, 8.2]	11.4 [5.7, 17.0]	9.3 [6.5, 12.0]	5.6 [3.4, 7.8]	2.8 [1.3, 4.3]
Burundi^a^^b^	89.1 [87.4, 90.8]	2.8 [0.84, 4.8]	27.8 [25.7, 29.8]	15.6 [5.1, 26.0]	16.1 [9.1, 23.2]	8.1 [4.2, 12.0]	5.7 [1.6, 9.8]	4.9 [1.6, 8.2]	2.3 [0.33, 4.3]
Cambodia^a^	77.6 [76.4, 78.9]	70.2 [68.9, 71.4]	40.0 [38.6, 41.5]	32.2 [16.5, 47.9]	27.9 [17.8, 38.0]	5.0 [2.7, 7.3]	14.7 [10.6, 18.8]	6.4 [4.2, 8.6]	8.3 [6.1, 10.6]
Cameroon^a^^b^	58.1 [56.7, 59.5]	44.5 [42.8, 46.1]	31.6 [30.1, 33.0]	20.9 [9.8, 32.0]	24.4 [6.8, 42.0]	23.5 [4.4, 42.5]	9.7 [6.7, 12.7]	12.4 [9.8, 15.0]	13.0 [10.0, 15.9]
Central African Republic^a^^b^	86.5 [84.0, 89.0]	56.3 [53.4, 59.2]	60.6 [57.1, 64.0]	25.3 [20.7, 29.9]	22.7 [13.9, 31.6]	18.9 [11.9, 25.8]	34.6 [31.2, 38.0]	28.3 [23.5, 33.1]	35.1 [30.1, 40.0]
Colombia	10.4 [9.6, 11.3]	10.5 [10.0, 11.0]	8.1 [7.6, 8.6]	7.8 [2.6, 13.0]	5.7 [1.4, 9.9]	3.5 [2.0, 5.0]	4.5 [−4.0, 13.0]	7.1 [5.3, 8.9]	1.8 [0.44, 3.2]
Congo, Dem. Rep.^a^^b^	87.9 [87.2, 88.6]	20.4 [17.9, 22.9]	14.1 [12.5, 15.8]	15.9 [11.4, 20.3]	9.6 [6.5, 12.7]	7.7 [4.9, 10.6]	31.9 [28.5, 35.4]	13.8 [7.6, 19.9]	15.7 [12.2, 19.2]
Cote d’Ivoire^a^^b^	71.7 [71.2, 72.2]	62.4 [61.2, 63.7]	40.6 [38.7, 42.6]	20.0 [12.0, 27.9]	22.8 [13.6, 32.1]	16.6 [8.7, 24.5]	13.5 [12.3, 14.8]	19.3 [17.0, 21.6]	13.5 [9.1, 18.0]
Dominican Republic	13.3 [12.6, 14.0]	12.3 [11.6, 13.0]	4.8 [3.9, 5.7]	1.1 [0.55, 1.7]	0.88 [0.47, 1.3]	0.29 [−0.13, 0.72]	8.4 [5.4, 11.4]	9.1 [6.7, 11.5]	3.5 [−1.2, 8.3]
Egypt^a^	5.4 [4.9, 5.9]	0.11 [−0.17, 0.40]	2.7 [2.4, 2.9]	18.6 [9.9, 27.4]	9.8 [4.9, 14.8]	3.7 [2.2, 5.2]	1.0 [0.55, 1.6]	1.3 [0.45, 2.1]	5.7 [2.8, 8.5]
Ethiopia^a^^b^	76.3 [75.3, 77.3]	55.8 [53.9, 57.7]	49.6 [47.7, 51.4]	60.6 [50.9, 70.4]	62.1 [52.3, 71.9]	58.4 [53.0, 63.8]	33.7 [28.4, 39.0]	18.4 [13.7, 23.0]	22.4 [18.6, 26.3]
Ghana^a^^b^	59.5 [57.7, 61.3]	25.6 [23.6, 27.7]	23.4 [21.6, 25.2]	31.1 [22.4, 39.9]	26.3 [13.0, 39.6]	15.5 [11.2, 19.8]	7.0 [4.1, 10.0]	2.3 [−0.23, 4.9]	0.18 [−2.3, 2.7]
Haiti^a^	**18.6 [15.5, 21.7]**	**50.4 [48.4, 52.5]**	**39.4 [37.6, 41.1]**	48.6 [31.6, 65.7]	41.3 [27.9, 54.7]	34.7 [18.1, 51.2]	9.7 [5.4, 14.0]	7.2 [3.4, 10.9]	−1.2 [−5.2, 2.8]
Indonesia^a^	16.5 [15.9, 17.1]	18.3 [17.7, 18.9]	12.3 [11.8, 12.8]	17.0 [11.5, 22.5]	14.5 [7.9, 21.1]	9.6 [4.1, 15.2]	9.6 [7.7, 11.5]	13.6 [11.8, 15.3]	10.1 [8.6, 11.6]
Jordan	8.9 [8.1, 9.7]	0.48 [0.22, 0.73]	0.0 [−0.04, 0.04]	0.28 [0.09, 0.46]	0.33 [0.20, 0.47]	0.18 [−0.13, 0.50]	−0.72 [−7.5, 6.1]	−0.01 [−1.7, 1.7]	0.85 [−0.10, 1.8]
Kenya^a^^b^	**14.8 [13.8, 15.9]**	**40.0 [38.4, 41.7]**	**33.4 [32.5, 34.2]**	28.0 [16.2, 39.7]	26.8 [18.5, 35.0]	22.2 [14.5, 29.8]	8.8 [5.5, 12.1]	2.4 [−0.15, 5.1]	3.9 [2.7, 5.1]
Lao PDR^a^	**24.6 [21.9, 27.4]**	**46.6 [44.9, 48.4]**	**37.8 [36.9, 38.6]**	21.0 [4.6, 37.5]	54.6 [36.8, 72.4]	38.5 [23.1, 54.0]	**10.0 [3.4, 16.5]**	**19.2 [13.2, 25.1]**	**19.9 [17.6, 22.3]**
Malawi^a^^b^	85.0 [82.4, 87.6]	41.7 [40.4, 43.0]	66.8 [65.1, 68.6]	12.5 [4.2, 20.8]	10.5 [3.0, 18.1]	2.6 [0.94, 4.4]	8.2 [6.4, 9.9]	2.6 [1.6, 3.6]	2.6 [1.7, 3.4]
Mali^a^^b^	**33.5 [32.0, 35.0]**	**53.4 [51.8, 55.0]**	**44.7 [43.1, 46.4]**	26.8 [11.8, 41.8]	17.7 [8.0, 27.4]	18.6 [13.3, 24.0]	22.0 [19.5, 24.6]	3.0 [0.69, 5.4]	8.3 [3.7, 12.9]
Moldova	66.3 [64.1, 68.5]	10.2 [8.7, 11.8]	12.9 [11.6, 14.2]		0.21 [−0.02, 0.44]	0.18 [−0.31, 0.69]	1.2 [−4.5, 7.0]	−1.8 [−4.8, 1.2]	0.86 [−5.7, 7.4]
Mongolia	76.6 [75.5, 77.7]	19.5 [18.4, 20.7]	14.3 [13.5, 15.2]	0.23 [−0.60, 1.0]	0.29 [0.00, 0.59]	0.23 [−0.19, 0.66]	**0.02 [−1**.**3, 1.3]**	**2.9 [0.92, 4.8]**	**10.7 [8.2, 13.1]**
Mozambique^a^^b^	97.4 [96.8, 98.0]	66.6 [65.6, 67.6]	46.5 [44.9, 48.2]	24.4 [16.1, 32.7]	17.2 [11.1, 23.4]	19.8 [13.4, 26.2]	14.5 [12.7, 16.3]	11.5 [9.9, 13.1]	7.5 [5.8, 9.1]
Namibia^b^	71.2 [69.5, 72.9]	63.9 [62.2, 65.5]	56.3 [53.7, 58.9]	10.5 [5.7, 15.3]	8.8 [4.1, 13.5]	5.2 [1.4, 8.9]	4.7 [1.6, 7.7]	5.4 [2.9, 7.8]	−1.4 [−4.2, 1.3]
Nepal^a^	88.3 [87.3, 89.3]	51.6 [49.8, 53.4]	6.4 [5.7, 7.1]	47.5 [33.5, 61.4]	45.0 [29.7, 60.3]	26.7 [16.7, 36.7]	8.2 [6.0, 10.5]	6.1 [4.2, 7.9]	−0.49 [−2.2, 1.2]
Niger^a^^b^	92.3 [90.0, 94.6]	82.1 [81.0, 83.2]	76.5 [75.2, 77.8]	31.4 [23.6, 39.2]	46.9 [36.3, 57.5]	31.7 [22.2, 41.2]		16.8 [13.2, 20.5]	11.8 [8.6, 15.1]
Nigeria^a^^b^	75.4 [73.7, 77.2]	24.4 [23.7, 25.2]	25.3 [24.6, 26.0]	36.6 [24.7, 48.5]	40.8 [25.5, 56.1]	40.3 [19.9, 60.7]	48.8 [40.3, 57.3]	39.2 [36.4, 42.0]	42.9 [40.8, 44.9]
Peru^a^	14.4 [13.8, 14.9]	11.4 [10.8, 12.1]	8.6 [8.0, 9.1]	18.5 [9.7, 27.3]	11.5 [5.2, 17.8]	8.7 [3.1, 14.2]	4.4 [2.6, 6.2]	6.1 [3.3, 9.0]	2.4 [0.31, 4.5]
Philippines^a^	12.8 [12.0, 13.6]	13.8 [12.9, 14.6]	10.4 [9.6, 11.1]	23.7 [11.1, 36.2]	23.4 [10.9, 35.9]	14.8 [7.2, 22.4]	7.8 [5.7, 9.9]	6.4 [4.7, 8.1]	6.2 [4.4, 8.0]
Rwanda^a^^b^	83.6 [82.5, 84.6]	57.8 [56.6, 59.1]	14.0 [12.7, 15.2]	25.9 [17.7, 34.2]	16.7 [7.3, 26.2]	2.5 [1.0, 4.1]	2.0 [0.08, 3.9]	0.44 [−1.1, 2.0]	5.1 [0.35, 9.9]
Sao Tome and Principe^a^^b^	66.1 [63.7, 68.4]	35.5 [32.0, 39.0]	38.1 [35.6, 40.6]	6.1 [0.76, 11.4]	4.2 [3.1, 5.3]	2.4 [0.87, 3.9]	3.0 [−1.3, 7.3]	6.7 [3.5, 10.0]	0.87 [−1.3, 3.1]
Senegal^a^^b^	57.0 [56.1, 57.9]	42.3 [41.1, 43.5]	32.5 [31.2, 33.9]	21.6 [14.7, 28.5]	28.7 [18.7, 38.6]	21.1 [14.5, 27.7]		0.87 [−1.1, 2.8]	2.6 [0.66, 4.7]
Sierra Leone^a^^b^	35.7 [29.8, 41.6]	32.0 [29.4, 34.7]	27.0 [25.3, 28.7]	17.2 [11.8, 22.6]	18.1 [9.2, 27.0]	9.1 [4.1, 14.2]	6.4 [1.4, 11.5]	2.5 [−3.9, 9.0]	−3.2 [−5.8, −0.69]
Suriname	18.0 [16.7, 19.4]	13.1 [11.8, 14.4]	13.7 [12.4, 14.9]	4.4 [−0.12, 9.0]	3.4 [1.3, 5.5]	3.5 [3.0, 4.1]			
Swaziland^a^^b^	53.6 [51.6, 55.6]	16.1 [14.5, 17.7]		9.9 [2.5, 17.3]	11.2 [4.7, 17.7]	7.0 [3.0, 10.9]	6.0 [3.1, 8.8]	−0.24 [−2.6, 2.1]	0.70 [−1.3, 2.7]
Togo^a^^b^	62.9 [61.6, 64.2]	56.3 [54.6, 58.0]	62.3 [60.0, 64.6]	22.8 [17.0, 28.6]	12.2 [9.7, 14.6]	27.1 [20.6, 33.7]	10.1 [6.6, 13.7]	10.1 [6.5, 13.8]	5.7 [2.8, 8.6]
Uganda^a^^b^	73.7 [70.0, 77.4]	50.3 [45.9, 54.7]	40.9 [37.9, 44.0]	−2.1 [−11.6, 7.3]	18.9 [8.5, 29.3]	13.9 [6.8, 20.9]	8.6 [4.8, 12.3]	5.2 [−14.3, 24.9]	3.8 [−1.2, 9.0]
Vietnam^a^	76.5 [75.8, 77.1]	50.2 [48.1, 52.4]	15.5 [14.3, 16.6]		12.2 [3.1, 21.4]	5.8 [−0.07, 11.8]	31.1 [29.1, 33.1]	13.8 [9.9, 17.8]	
Zambia^a^^b^	80.1 [78.8, 81.4]	51.8 [50.0, 53.6]	35.6 [34.3, 36.9]	31.9 [23.1, 40.6]	26.7 [20.2, 33.2]	14.7 [9.3, 20.0]	5.6 [3.5, 7.7]	−3.5 [−11.9, 4.7]	5.4 [4.0, 6.9]

^a^Countdown country; ^b^Sub-Saharan African country.

Countries whose concentration index became significantly father away from zero between round 1 and round 3 are shown in bold.

**Table A12.** Ratio of Q5 to Q1 in the three child health outcomes by survey round and country, 41 countries.

	Infant mortality per 1,000 live births			Under-five mortality per 1,000 live births			Stunting prevalence (%)		
Country	Round 1	Round 2	Round 3	Round 1	Round 2	Round 3	Round 1	Round 2	Round 3
Armenia	0.49 [0.26, 0.72]	0.33 [0.11, 0.55]	0.98 [0.23, 1.73]	0.46 [0.25, 0.67]	0.45 [0.18, 0.72]	0.84 [0.22, 1.46]	0.58 [0.40, 0.77]	0.92 [0.45, 1.39]	0.71 [0.44, 0.98]
Bangladesh^a^	0.71 [0.58, 0.84]	0.54 [0.38, 0.69]	0.55 [0.30, 0.81]	0.58 [0.48, 0.69]	0.50 [0.37, 0.63]	0.56 [0.31, 0.81]	0.49 [0.43, 0.54]	0.49 [0.41, 0.56]	0.40 [0.33, 0.47]
Benin^a^^b^	0.44 [0.32, 0.56]	0.65 [0.54, 0.76]	0.57 [0.43, 0.72]	0.46 [0.37, 0.56]	0.55 [0.47, 0.62]	0.50 [0.40, 0.61]	0.54 [0.45, 0.62]	0.57 [0.53, 0.61]	0.76 [0.68, 0.84]
Burkina Faso^a^^b^	0.80 [0.61, 0.98]	0.59 [0.48, 0.70]	0.70 [0.58, 0.81]	0.54 [0.46, 0.62]	0.50 [0.44, 0.57]	0.47 [0.36, 0.58]	0.42 [0.36, 0.48]		
Burundi^a^^b^			0.53 [0.40, 0.65]		0.54 [0.44, 0.64]	0.67 [0.60, 0.74]	0.59 [0.53, 0.66]		
Cambodia^a^	0.45 [0.35, 0.55]	0.34 [0.25, 0.43]	0.25 [0.09, 0.41]	0.40 [0.32, 0.47]	0.34 [0.26, 0.42]	0.25 [0.10, 0.39]	0.54 [0.45, 0.63]	0.43 [0.36, 0.51]	0.44 [0.36, 0.51]
Cameroon^a^^b^	0.50 [0.38, 0.63]	0.56 [0.45, 0.68]	0.45 [0.37, 0.54]	0.39 [0.33, 0.45]	0.33 [0.25, 0.40]	0.32 [0.26, 0.38]	0.25 [0.20, 0.30]		
Central African Republic^a^^b^						**0.82 [0.78, 0.85]**	**0.63 [0.55, 0.71]**	**0.66 [0.59, 0.73]**	
Colombia	0.54 [0.32, 0.75]	0.45 [0.28, 0.62]	0.55 [0.37, 0.73]	0.51 [0.31, 0.70]	0.40 [0.25, 0.54]	0.43 [0.30, 0.57]	0.33 [0.25, 0.41]	0.17 [0.14, 0.21]	0.33 [0.28, 0.39]
Congo, Dem. Rep.^a^^b^	0.51 [0.36, 0.66]	0.78 [0.61, 0.95]	0.52 [0.41, 0.63]	0.65 [0.53, 0.77]	**0.56 [0.53, 0.58]**	**0.53 [0.39, 0.68]**	**0.46 [0.39, 0.52]**		
Cote d’Ivoire^a^^b^		0.70 [0.48, 0.93]		0.66 [0.50, 0.83]	0.57 [0.48, 0.65]	0.36 [0.27, 0.45]			
Dominican Republic	0.40 [0.26, 0.54]	0.58 [0.36, 0.80]	0.35 [0.04, 0.65]	0.34 [0.23, 0.46]	0.51 [0.32, 0.69]	0.40 [0.12, 0.68]	0.19 [0.13, 0.25]	0.26 [0.19, 0.33]	0.34 [0.13, 0.55]
Egypt^a^	0.38 [0.30, 0.46]	0.39 [0.27, 0.52]	0.5 [0.32, 0.67]	0.34 [0.27, 0.40]	0.38 [0.27, 0.49]	0.45 [0.30, 0.60]	0.48 [0.42, 0.54]	0.91 [0.82, 1.00]	0.97 [0.94, 1.00]
Ethiopia^a^^b^	**1.02 [0.83, 1.21]**	**0.75 [0.59, 0.92]**	**0.65 [0.52, 0.79]**	**0.92 [0.77, 1.07]**	**0.71 [0.58, 0.83]**	**0.61 [0.49, 0.74]**	**0.80 [0.72, 0.89]**	**0.76 [0.64, 0.88]**	**0.59 [0.52, 0.66]**
Ghana^a^^b^	0.92 [0.63, 1.21]	0.77 [0.47, 1.07]	0.92 [0.53, 1.31]	0.68 [0.49, 0.86]	0.58 [0.39, 0.77]	0.69 [0.44, 0.94]	0.36 [0.28, 0.45]	0.42 [0.30, 0.55]	0.34 [0.16, 0.51]
Haiti^a^	0.99 [0.69, 1.29]	0.56 [0.39, 0.74]	0.81 [0.60, 1.03]	0.66 [0.51, 0.82]	0.44 [0.31, 0.57]	0.59 [0.45, 0.73]	0.27 [0.20, 0.33]	0.17 [0.11, 0.24]	0.21 [0.14, 0.27]
Indonesia^a^	0.28 [0.20, 0.35]	0.46 [0.33, 0.59]	0.32 [0.24, 0.40]	0.28 [0.21, 0.35]	0.39 [0.29, 0.49]	0.31 [0.24, 0.38]			
Jordan	0.64 [0.34, 0.94]	0.98 [0.45, 1.50]	0.32 [0.09, 0.55]	0.65 [0.37, 0.93]	0.90 [0.43, 1.37]	0.40 [0.14, 0.66]	**0.36 [0.25, 0.48]**	**0.50 [0.32, 0.67]**	**0.12 [0.08, 0.17]**
Kenya^a^^b^	0.64 [0.48, 0.81]	0.87 [0.60, 1.14]	0.95 [0.47, 1.43]	0.61 [0.47, 0.75]	0.71 [0.50, 0.91]	0.82 [0.41, 1.23]	**0.56 [0.48, 0.64]**	**0.57 [0.46, 0.67]**	**0.38 [0.33, 0.42]**
Lao PDR^a^							**0.74 [0.63, 0.85]**	**0.40 [0.32, 0.47]**	**0.32 [0.28, 0.36]**
Malawi^a^^b^	0.60 [0.47, 0.74]		0.60 [0.50, 0.71]		0.62 [0.56, 0.68]	0.76 [0.71, 0.81]	0.64 [0.59, 0.68]		
Mali^a^^b^	0.65 [0.55, 0.75]	0.64 [0.55, 0.73]	0.72 [0.54, 0.90]	0.59 [0.51, 0.67]	0.52 [0.45, 0.59]	0.54 [0.42, 0.66]	0.48 [0.40, 0.55]	0.49 [0.44, 0.54]	0.45 [0.38, 0.51]
Moldova		0.79 [0.31, 1.27]		0.61 [0.27, 0.95]		0.45 [0.28, 0.63]	0.24 [0.08, 0.40]		
Mongolia							**0.37 [0.33, 0.40]**	**0.61 [0.50, 0.71]**	**0.27 [0.23, 0.31]**
Mozambique^a^^b^	0.49 [0.41, 0.58]	0.77 [0.62, 0.91]	0.54 [0.47, 0.62]	0.69 [0.58, 0.81]	0.46 [0.40, 0.52]	0.50 [0.45, 0.56]	0.47 [0.42, 0.52]		
Namibia^b^	0.53 [0.26, 0.80]	0.39 [0.24, 0.55]	0.43 [0.20, 0.65]	0.49 [0.26, 0.72]	0.32 [0.21, 0.43]	0.46 [0.26, 0.65]	0.42 [0.27, 0.57]	0.32 [0.21, 0.43]	0.27 [0.11, 0.44]
Nepal^a^	0.62 [0.47, 0.76]	0.55 [0.35, 0.76]	0.51 [0.40, 0.62]	0.48 [0.32, 0.63]	**0.62 [0.56, 0.67]**	**0.49 [0.43, 0.55]**	**0.27 [0.23, 0.32]**		
Niger^a^^b^		0.74 [0.59, 0.88]	0.81 [0.63, 0.99]	0.75 [0.63, 0.87]	0.77 [0.65, 0.90]	0.75 [0.67, 0.82]	0.69 [0.60, 0.79]	0.74 [0.64, 0.84]	
Nigeria^a^^b^	0.39 [0.28, 0.49]	0.58 [0.50, 0.65]	0.52 [0.45, 0.59]	0.31 [0.24, 0.38]	0.40 [0.35, 0.44]	0.38 [0.33, 0.43]	0.38 [0.30, 0.45]	0.45 [0.42, 0.48]	0.33 [0.30, 0.36]
Peru^a^	0.11 [−0.02, 0.24]	0.27 [0.14, 0.39]	0.51 [0.25, 0.77]	0.13 [0.00, 0.26]	0.33 [0.20, 0.46]	0.4 [0.20, 0.59]	0.08 [0.02, 0.15]	0.08 [0.05, 0.11]	0.09 [0.05, 0.12]
Philippines^a^	0.47 [0.29, 0.64]	0.36 [0.22, 0.51]	0.34 [0.21, 0.48]	0.31 [0.20, 0.42]	0.29 [0.18, 0.39]	0.30 [0.19, 0.40]			
Rwanda^a^^b^	0.63 [0.51, 0.75]	0.64 [0.52, 0.75]	0.50 [0.24, 0.75]	0.62 [0.53, 0.72]	0.57 [0.48, 0.65]	0.47 [0.26, 0.68]	**0.60 [0.54, 0.66]**	**0.57 [0.50, 0.65]**	**0.43 [0.35, 0.50]**
Sao Tome and Principe^a^^b^	0.51 [0.18, 0.85]		0.32 [0.12, 0.52]	0.47 [0.35, 0.60]	0.51 [0.32, 0.69]	0.36 [0.22, 0.50]			
Senegal^a^^b^		0.45 [0.33, 0.57]	0.44 [0.20, 0.68]	0.35 [0.28, 0.42]	0.30 [0.15, 0.45]	0.43 [0.38, 0.48]	0.26 [0.16, 0.36]	0.29 [0.19, 0.38]	
Sierra Leone^a^^b^		0.62 [0.46, 0.77]	0.87 [0.71, 1.03]	0.68 [0.54, 0.81]	0.77 [0.65, 0.89]	0.65 [0.55, 0.76]	0.63 [0.46, 0.79]	0.66 [0.55, 0.77]	
Suriname							0.19 [0.05, 0.33]	0.26 [0.15, 0.37]	0.41 [0.24, 0.58]
Swaziland^a^^b^		0.98 [0.66, 1.30]		0.84 [0.61, 1.07]	0.42 [0.33, 0.51]	0.46 [0.34, 0.58]	0.33 [0.25, 0.41]		
Togo^a^^b^			0.52 [0.31, 0.73]		0.38 [0.26, 0.50]	0.51 [0.40, 0.61]	0.31 [0.23, 0.39]		
Uganda^a^^b^	0.95 [0.76, 1.14]	0.63 [0.49, 0.76]	0.63 [0.46, 0.79]	**0.92 [0.76, 1.08]**	**0.67 [0.56, 0.78]**	**0.57 [0.45, 0.69]**	**0.94 [0.83, 1.0]**	**0.57 [0.45, 0.68]**	**0.60 [0.43, 0.77]**
Vietnam^a^							0.33 [0.22, 0.44]		
Zambia^a^^b^	0.48 [0.36, 0.61]	1.08 [0.86, 1.30]	0.70 [0.46, 0.95]	0.47 [0.37, 0.57]	0.87 [0.72, 1.02]	0.58 [0.41, 0.74]	0.58 [0.51, 0.65]	0.70 [0.58, 0.82]	0.60 [0.55, 0.64]

^a^Countdown country; ^b^Sub-Saharan African country.

Countries whose ratio of Q5 to Q1 became significantly farther away from one between round 1 and round 3 are shown in bold.

**Table A13.** Ratio of Q5 to Q1 in the three selected child health interventions by survey round and country, 41 countries.

	Access to improved sanitation (%)			Delivered with SBA (%)			Received full immunization (%)		
Country	Round 1	Round 2	Round 3	Round 1	Round 2	Round 3	Round 1	Round 2	Round 3
Armenia	**0.99 [0.97, 1.0]**	**1.2 [1.2, 1.3]**	**3.8 [3.7, 4.0]**	1.0 [1.0, 1.0]	1.0 [0.98, 1.0]	1 [0.97, 1.0]	1.0 [0.72, 1.2]	1.0 [0.39, 1.6]	1.0 [0.72, 1.3]
Bangladesh^a^	3.6 [3.5, 3.8]	4.7 [4.5, 5.0]	2.6 [2.5, 2.7]	12.3 [8.7, 15.9]	8.2 [6.1, 10.4]	4.2 [3.3, 5.1]	1.5 [1.2, 1.7]	1.1 [0.99, 1.2]	1.3 [1.1, 1.4]
Benin^a^^b^	89.8 [71.6, 108.0]	78.3 [61.4, 95.3]	86.9 [69.0, 104.8]	1.9 [1.6, 2.1]	1.6 [1.5, 1.7]	1.5 [1.4, 1.6]	1.4 [1.1, 1.7]	1.9 [1.6, 2.1]	1.5 [1.3, 1.8]
Burkina Faso^a^^b^	90.8 [72.6, 109.1]	4.1 [0.26, 8.1]	87.8 [69.8, 105.7]	2.3 [2.0, 2.7]	1.1 [0.98, 1.3]	1.8 [1.7, 2.0]	1.7 [1.4, 2.1]	1.2 [0.99, 1.5]	1.1 [1.0, 1.2]
Burundi^a^^b^	21.2 [12.3, 30.0]	1.1 [1.0, 1.2]	4.9 [4.6, 5.3]	2.3 [0.75, 3.8]	2.1 [1.8, 2.5]	1.5 [1.3, 1.6]	1.3 [1.0, 1.7]	1.1 [0.86, 1.4]	1.0 [0.97, 1.1]
Cambodia^a^	82.5 [65.1, 99.9]	82.4 [65.0, 99.8]	13.8 [12.8, 14.8]	5.7 [4.6, 6.8]	4.1 [3.4, 4.7]	1.2 [0.91, 1.6]	2.3 [1.8, 2.9]	1.3 [1.1, 1.5]	1.4 [1.3, 1.6]
Cameroon^a^^b^	79.6 [62.5, 96.8]	11.5 [10.4, 12.6]	4.9 [4.7, 5.1]	**3.1 [2.5, 3.7]**	**5.0 [3.7, 6.2]**	**5.1 [4.3, 6.0]**	1.6 [1.3, 2.0]	1.5 [1.2, 1.8]	2.1 [1.7, 2.5]
Central African Republic^a^^b^	7.2 [2.0, 12.4]	15.3 [7.8, 22.9]	16.8 [8.9, 24.6]	3.2 [2.7, 3.7]	3.3 [2.6, 3.9]	2.6 [2.2, 3.0]	4.6 [2.1, 7.0]	3.1 [1.9, 4.2]	4.7 [3.0, 6.4]
Colombia	1.8 [1.7, 1.8]	1.7 [1.7, 1.8]	1.4 [1.4, 1.5]	1.5 [1.3, 1.6]	1.3 [1.2, 1.3]	1.1 [1.1, 1.1]	1.3 [1.0, 1.6]	1.5 [1.2, 1.7]	1.0 [0.92, 1.1]
Congo, Dem. Rep.^a^^b^	40.8 [28.5, 53.1]	3.0 [2.8, 3.2]	2.3 [2.2, 2.4]	2.0 [1.8, 2.1]	1.5 [1.4, 1.7]	1.4 [1.3, 1.5]	5.0 [3.7, 6.3]	2.5 [1.7, 3.3]	1.7 [1.4, 2.0]
Cote d’Ivoire^a^^b^	97.8 [78.8, 116.7]	70.2 [54.2, 86.3]	16.7 [15.2, 18.2]	2.7 [2.6, 2.9]	3.3 [2.6, 3.9]	2.4 [2.1, 2.8]	1.8 [1.7, 1.9]	1.5 [1.3, 1.8]	1.7 [1.3, 2.0]
Dominican Republic	1.8 [1.8, 1.9]	1.8 [1.7, 1.8]	1.2 [1.2, 1.3]	1.0 [1.0, 1.0]	1.0 [1.0, 1.0]	1.0 [0.99, 1.0]	1.3 [0.99, 1.7]	1.5 [1.3, 1.7]	1.3 [0.69, 2.0]
Egypt^a^	1.3 [1.3, 1.4]	1.0 [0.99, 1.0]	1.1 [1.1, 1.1]	2.7 [2.4, 2.9]	1.6 [1.5, 1.7]	1.2 [1.1, 1.2]	1.0 [0.95, 1.0]	1.0 [1.0, 1.0]	1.0 [−0.0, 2.1]
Ethiopia^a^^b^	59.4 [44.6, 74.2]	29.7 [19.2, 40.1]	17.4 [13.8, 21.0]	23 [13.7, 32.2]	22.3 [14.6, 30.1]	17.3 [11.1, 23.6]	4.5 [2.7, 6.2]	2.4 [1.5, 3.4]	3.0 [2.0, 4.0]
Ghana^a^^b^	74.7 [58.2, 91.3]	4.7 [4.5, 4.9]	4.3 [4.1, 4.5]	4.6 [3.7, 5.5]	4.1 [3.3, 4.8]	2.0 [1.7, 2.3]	1.4 [1.1, 1.7]	1.1 [0.92, 1.2]	0.98 [0.80, 1.1]
Haiti^a^	**2.9 [2.5, 3.3]**	**64.8 [49.4, 80.3]**	**12.5 [11.6, 13.4]**	18.2 [12.5, 24.0]	9.0 [6.3, 11.6]	7.5 [6.1, 8.9]	1.6 [0.89, 2.4]	1.6 [1.1, 2.1]	0.99 [0.70, 1.2]
Indonesia^a^	2.3 [2.2, 2.4]	2.5 [2.4, 2.6]	1.8 [1.8, 1.9]	2.2 [2.0, 2.4]	2.0 [1.9, 2.2]	1.6 [1.5, 1.6]	1.7 [1.4, 2.0]	1.8 [1.6, 2.1]	1.6 [1.5, 1.8]
Jordan	1.5 [1.5, 1.6]	1.0 [0.99, 1.0]	1.0 [0.98, 1.0]	1.0 [1.0, 1.0]	1.0 [1.0, 1.0]	1.0 [1.0, 1.0]	1.1 [0.71, 1.6]	1.0 [0.96, 1.2]	1.0 [0.81, 1.2]
Kenya^a^^b^	**2.2 [2.2, 2.3]**	**7.6 [7.1, 8.1]**	**5.3 [5.0, 5.6]**	4.4 [3.5, 5.3]	3.6 [2.9, 4.4]	2.9 [2.9, 3.0]	1.5 [1.1, 1.8]	1.1 [0.92, 1.3]	1.3 [1.1, 1.5]
Lao PDR^a^	**4.0 [3.2, 4.8]**	**14.1 [13.1, 15.2]**	**9.5 [8.9, 10.0]**	**3.1 [2.7, 3.4]**	**27.0 [16.5, 37.6]**	**8.3 [6.8, 9.9]**	1.5 [0.88, 2.1]	2.4 [1.4, 3.4]	2.0 [1.6, 2.4]
Malawi^a^^b^	15.3 [7.8, 22.8]	10.1 [8.6, 11.6]	32.8 [21.8, 43.8]	1.9 [1.7, 2.0]	1.8 [1.6, 1.9]	1.1 [1.1, 1.1]	1.4 [1.2, 1.7]	1.1 [1.0, 1.2]	1.1 [1.0, 1.1]
Mali^a^^b^	**7.2 [6.2, 8.2]**	**26.3 [22.1, 30.4]**	**15.8 [14.5, 17.1]**	3.8 [3.0, 4.6]	2.3 [2.0, 2.7]	2.5 [2.1, 2.8]	2.8 [2.0, 3.7]	1.1 [0.95, 1.3]	1.6 [1.2, 2.0]
Moldova	[80.8, 119.1]	1.7 [1.6, 1.7]	1.9 [1.8, 1.9]		1.0 [0.99, 1.0]	1.0 [1.0, 1.0]	1.0 [0.66, 1.3]	2.0 [1.0, 2.9]	1.1 [0.65, 1.6]
Mongolia	99.0 [79.9, 118.1]	3.5 [3.4, 3.7]	2.9 [2.8, 3.0]	1.0 [0.98, 1.0]	1.0 [1.0, 1.0]	1.0 [1.0, 1.0]	**1.0 [0.94, 1.0]**	**1.0 [0.85, 1.2]**	**1.9 [1.6, 2.2]**
Mozambique^a^^b^	**9.6 [3.7, 15.6]**	**68.6 [52.7, 84.5]**	**50.6 [37.0, 64.3]**	3.4 [2.9, 3.9]	2.4 [2.0, 2.7]	2.6 [2.2, 3.0]	1.9 [1.6, 2.2]	1.6 [1.4, 1.8]	1.3 [1.1, 1.5]
Namibia^b^	99.4 [80.3, 118.5]	98.7 [79.7, 117.8]	85.6 [69.9, 101.3]	1.7 [1.4, 1.9]	1.6 [1.4, 1.7]	1.3 [1.3, 1.3]	1.2 [0.96, 1.5]	1.3 [1.0, 1.6]	0.67 [0.61, 0.74]
Nepal^a^	56.3 [41.9, 70.7]	29.6 [26.2, 32.9]	1.2 [1.2, 1.3]	11.6 [8.2, 15.0]	11.6 [7.2, 16.0]	3.8 [3.3, 4.4]	1.5 [1.2, 1.7]	1.3 [1.1, 1.5]	0.99 [0.89, 1.0]
Niger^a^^b^	**8.2 [2.7, 13.7]**	**39.2 [27.2, 51.2]**	**78.0 [61.0, 94.9]**	3.8 [2.9, 4.8]	11.2 [8.0, 14.4]	5.2 [4.2, 6.2]		2.4 [1.6, 3.1]	1.9 [1.5, 2.2]
Nigeria^a^^b^	61.0 [46.0, 76.0]	3.4 [3.2, 3.5]	3.5 [3.4, 3.6]	**7.0 [4.9, 9.1]**	**9.7 [8.1, 11.3]**	**14.1 [11.6, 16.5]**	11.7 [5.2, 18.2]	11.0 [8.6, 13.4]	15.2 [11.4, 19.0]
Peru^a^	2.2 [2.2, 2.3]	1.7 [1.6, 1.7]	1.4 [1.4, 1.5]	2.5 [2.4, 2.6]	1.7 [1.6, 1.9]	1.5 [1.4, 1.6]	1.2 [0.74, 1.7]	1.1 [0.87, 1.4]	1.0 [0.91, 1.2]
Philippines^a^	1.8 [1.8, 1.9]	1.9 [1.9, 2.0]	1.6 [1.6, 1.6]	3.5 [3.0, 3.9]	3.4 [3.0, 3.9]	2.0 [1.9, 2.2]	1.4 [1.3, 1.6]	1.3 [1.2, 1.5]	1.2 [1.1, 1.4]
Rwanda^a^^b^	41.8 [29.4, 54.2]	67.7 [51.9, 83.4]	2.0 [1.9, 2.0]	3.0 [2.4, 3.6]	2.3 [2.0, 2.6]	1.1 [1.1, 1.1]	1.0 [0.95, 1.2]	0.99 [0.89, 1.0]	1.0 [0.13, 2.0]
Sao Tome and Principe^a^^b^	71.8 [55.5, 88.0]	7.7 [7.2, 8.3]	7.4 [7.0, 7.8]	1.2 [0.91, 1.5]	1.2 [1.0, 1.3]	1.1 [1.0, 1.2]	1.1 [0.82, 1.4]	1.1 [0.80, 1.5]	1.0 [0.95, 1.1]
Senegal^a^^b^	97.4 [78.5, 116.3]	12.1 [11.2, 13.0]	10.0 [9.3, 10.6]	2.8 [2.5, 3.1]	4.3 [3.6, 4.9]	2.8 [2.5, 3.0]		1.1 [0.91, 1.2]	1.1 [0.92, 1.2]
Sierra Leone^a^^b^	5.0 [3.9, 6.0]	6.6 [6.3, 7.0]	4.8 [4.6, 5.1]	2.3 [1.8, 2.9]	2.5 [2.1, 3.0]	1.5 [1.4, 1.7]	1.5 [1.1, 1.9]	1.0 [0.75, 1.3]	0.84 [0.70, 0.98]
Suriname	3.2 [3.1, 3.3]	1.9 [1.9, 2.0]	1.8 [1.8, 1.9]	1.3 [1.1, 1.4]	1.1 [1.0, 1.2]	1.1 [1.0, 1.2]			
Swaziland^a^^b^	92.6 [74.1, 111.0]	2.5 [2.4, 2.6]		1.8 [1.5, 2.0]	1.8 [1.5, 2.0]	1.4 [1.2, 1.6]	1.3 [1.1, 1.5]	0.95 [0.79, 1.1]	0.90 [0.76, 1.0]
Togo^a^^b^	92.4 [74.0, 110.9]	67.2 [55.0, 79.3]	39.7 [34.6, 44.8]	2.9 [2.2, 3.7]	1.7 [1.5, 1.9]	3.5 [3.3, 3.7]	1.9 [1.7, 2.0]	1.5 [1.2, 1.9]	1.1 [1.0, 1.2]
Uganda^a^^b^	13.1 [6.1, 20.0]	8.6 [7.9, 9.4]	17.4 [15.5, 19.3]	**1.1 [0.82, 1.5]**	**2.5 [2.2, 2.8]**	**2.0 [1.7, 2.2]**	0.82 [0.60, 1.0]	1.1 [0.92, 1.3]	1.0 [0.87, 1.2]
Vietnam^a^	88.4 [70.4, 106.4]	16.9 [15.5, 18.3]	2.7 [2.6, 2.8]		1.8 [1.4, 2.3]	1.3 [1.3, 1.4]	7.2 [4.4, 10.0]	2.1 [1.6, 2.6]	
Zambia^a^^b^	76.5 [59.8, 93.3]	49.7 [42.7, 56.6]	6.8 [6.4, 7.1]	4.5 [3.7, 5.3]	3.4 [2.9, 4.0]	2.0 [1.9, 2.1]	1.2 [1.0, 1.4]	1.1 [0.97, 1.2]	1.2 [1.2, 1.3]

^a^Countdown country; ^b^Sub-Saharan African country.

Countries whose ratio of Q5 to Q1 became significantly farther away from one between round 1 and round 3 are shown in bold.

For all three selected indicators, the poorest wealth quintile (Q1) has a substantial proportion of values as zero, leading to infinite large ratio when calculating Q5 to Q1. To avoid this problem, we replaced the extremely small values (less than 1%) with 1%.

## Appendix Method. Calculation methods of 95% CI in various tables/figures

Tables 1 and A6 are at the aggregate level. We took two steps to generate the mean estimate and the 95% confidence intervals for each indicator: First, for each country with available data, we calculated the point estimates of all indexes (overall value, Q1, Q5, difference between Q5 and Q1, concentration index, and ratio of Q5 to Q1) following the method of O’Donnell et al. (O’Donnell O, Wagstaff A. Analyzing health equity using household survey data: a guide to techniques and their implementation. World Bank Publications, 2007). Second, we treated each country as a subject to calculate the mean values. Then we generated standard errors for the point estimates and calculated 95% CI based on the point estimate and the standard errors.

Table A7 is generated in the same way as Table A6, yet instead of treating each country as an identical subject, we weighted the countries with their population sizes.

Tables A8 and A9 present absolute differences between Q5 and Q1 with the 95% CIs at the country level. To generate the 95% CI of the absolute difference, we used the formula $(x_{1}-x_{2})\pm Z_{{\left(1-\mu /2\right)}^{*}}\sqrt{\frac{\sigma_{1}^{2}}{n_{1}}+\frac{\sigma_{2}^{2}}{n_{2}}}$ . In this study, $x_{1}$ and $x_{2}$ represent the point estimates for Q5 and Q1 groups, $\sigma_{1}$ and $\sigma_{2}$ are the standard errors of the Q5 and Q1 groups, $n_{1}$ and $n_{2}$ are the observation numbers of the Q5 and Q1 groups.

Tables A10 and A11 present concentration indexes with the 95% CIs at the country level. For each country, we generated the 95% CI following exactly the World Bank instruction. Detailed information can be found below: http://siteresources.worldbank.org/INTPAH/Resources/Publications/459843-1195594469249/HealthEquityCh8.pdf (Accessed on March 2nd, 2017)

Tables A12 and A13 present ratios of Q5 to Q1 (Q5/Q1) with the 95% CIs at the country level. To generate the 95% CI, we used Taylor Expansion, using the formula $x_{1}/x_{2}\pm z_{\left(1-\mu /2\right)}*\sqrt{\frac{\sigma_{1}^{2}}{x_{2}^{2}}-2*\frac{x_{1}}{x_{2}^{3}}*COV(x_{1},x_{2})+\frac{x_{1}^{2}}{x_{2}^{4}}*\sigma_{2}^{2}}$ . In this study, $x_{1}$ and $x_{2}$ represent the point estimates for Q5 and Q1 groups, $\sigma_{1}$ and $\sigma_{2}$ are the standard deviation of the Q5 and Q1 groups, $n_{1}$ and $n_{2}$ are the observation numbers of the Q5 and Q1 groups.
