# Supplementary material for: Assessing levels and trends of child health inequality in 88 developing countries: from 2000 to 2014
Source: Glob Health Action. 2017 Dec 13;10(1):1408385. doi: 10.1080/16549716.2017.1408385 (PMC5727456; doi:10.1080/16549716.2017.1408385)
Supplement: Supplementary material [file ZGHA_A_1408385_SM3828.docx]

The underlying research materials for this article can be accessed at https://dhsprogram.com, https://www.unicef.org/statistics/index_

24302.html, <http://www.who.int/gho/health_equity/en/>
